# Autoimmunity and Carcinogenesis: Their Relationship under the Umbrella of Autophagy

**DOI:** 10.3390/biomedicines11041130

**Published:** 2023-04-08

**Authors:** Györgyi Műzes, Ferenc Sipos

**Affiliations:** Immunology Division, Department of Internal Medicine and Hematology, Semmelweis University, 1088 Budapest, Hungary; muzes.gyorgyi@med.semmelweis-univ.hu

**Keywords:** autophagy, autoimmunity, carcinogenesis, immune system, autoimmune disorders, tumor, treatment, tumor microenvironment

## Abstract

The immune system and autophagy share a functional relationship. Both innate and adaptive immune responses involve autophagy and, depending on the disease’s origin and pathophysiology, it may have a detrimental or positive role on autoimmune disorders. As a “double-edged sword” in tumors, autophagy can either facilitate or impede tumor growth. The autophagy regulatory network that influences tumor progression and treatment resistance is dependent on cell and tissue types and tumor stages. The connection between autoimmunity and carcinogenesis has not been sufficiently explored in past studies. As a crucial mechanism between the two phenomena, autophagy may play a substantial role, though the specifics remain unclear. Several autophagy modifiers have demonstrated beneficial effects in models of autoimmune disease, emphasizing their therapeutic potential as treatments for autoimmune disorders. The function of autophagy in the tumor microenvironment and immune cells is the subject of intensive study. The objective of this review is to investigate the role of autophagy in the simultaneous genesis of autoimmunity and malignancy, shedding light on both sides of the issue. We believe our work will assist in the organization of current understanding in the field and promote additional research on this urgent and crucial topic.

## 1. Introduction

The immune system is self-tolerant toward its own tissues, but defends against non-self and potentially harmful antigens (e.g., pathogens and tumor cells). Self-tolerance is shaped and regulated by multiple mechanisms, including the removal of autoreactive receptors in the bone marrow and thymus (i.e., central tolerance). Nevertheless, not every autoreactive cell is eliminated from primary lymphoid organs. Up to forty percent of the naïve T cell repertoire leaving the thymus is composed of low-affinity self-reactive T cells, which have the potential to trigger an autoaggressive immune response. A number of mechanisms have therefore evolved to avoid their activation during peripheral tolerance [1]. To maintain the equilibrium between tolerance and activation, specialized cells, such as Treg and Breg cells, tolerogenic DCs, and M2-polarized macrophages, are essential. This highly regulated condition can be disrupted by genetic pre-disposition, epigenetic alterations, and environmental variables, ultimately leading to the development of autoimmunity. Increasing immunological tolerance may, therefore, be a potential treatment for autoimmune disorders. However, tumor cells can also exploit the cells and systems involved in immunological tolerance to make the immune system tolerate the appearance of tumors [2]. Cancer cells are able to evade the self-protecting immune actions by attracting tolerogenic immune cells. Immune checkpoint proteins, poor antigen presentation, EMT, and altered RNA editing are characteristics of tumor cells. This problem could be related to the absence of tumor-specific or tumor-associated antigens that stimulate an immune response against cancer cells [3,4].

Autophagy is a cellular survival mechanism that degrades unused organelles, proteins, and pathogenic pathogens to preserve homeostasis [5]. Three forms of autophagy exist: macroautophagy, chaperone-mediated autophagy, and microautophagy. Macroautophagy has received the greatest attention and is often referred to as autophagy. Diverse PRRs are implicated in the onset of autophagy; however, this process is also blocked by Th2 cytokines, Bcl-2 and the canonical nutrient-sensing insulin-AKT-mTOR pathway [6]. It is, therefore, not unexpected that autophagy is engaged in both innate and adaptive immune responses and may play a harmful and/or therapeutic function in autoimmune disorders, depending on the disease’s etiology and the development of its major players [7].

Since autophagy aids in preventing chronic cell damage, it may inhibit the transition of cells into cancer-causing cells [8]. In conjunction with immune surveillance, autophagy possesses cancer-preventive properties. A reduction in autophagy has been connected with Treg cell infiltration, thereby lowering the efficacy of immune surveillance; this may facilitate the beginning of more tumors [9]. In turn, an increase in autophagy following the complete development of malignant tumors may facilitate the survival and expansion of tumor cells [8,10]. Autophagy may have both autonomous and non-autonomous tumor-promoting effects [11]; it is known that autophagy (and its defects) can affect the behavior of tumor cells [12,13].

The link between autoimmunity and carcinogenesis is not completely understood. As a critical mechanism between the two phenomena, autophagy could play an important role, though the specific details are not well understood. The objective of this review is to investigate the role of autophagy in the development of autoimmunity and cancer simultaneously, shedding light on both aspects of the issue. We believe that our work will facilitate the organization of current understanding in the field and encourage further study on this timely and important topic.

## 2. Autophagy and the Immune System

The autophagy machinery modulates the immune system. Activation of innate immune receptors, including TLRs and NLRs, can enhance autophagy [14]. TLRs can activate TRIF/RIP1/p38MAPK, JNK, and ERK signaling pathways or induce autophagy through MyD88 [15,16]. NLRs trigger autophagy directly via recruitment and interaction with ATG16L1 [17]. Autophagy activation enhances the recruitment of ATG8/LC3 to the phagosome membrane, the fusion of phagosomes with lysosomes, and the alteration of phagosomal content, thereby enhancing antigen presentation and adaptive immunity [18].

There is a functional connection between autophagy and immune cells. Autophagy activation can either promote or inhibit tumor formation by modulating the homeostasis, activation, proliferation, and differentiation of immune cells [19]. Autophagy induced by mTOR inhibition enhances CD8+ T cell differentiation into CTLs, whereas mTOR activation promotes T cell differentiation into Th cells [20]. Through boosting antigen presentation, autophagy promotes DC and B cell growth, plasma cell differentiation, and the production of specific IgM and IgG molecules [21]. The interaction between cytosolic phosphorylated FoxO1 and Atg7 induces autophagy and promotes the development of NKT cells with antitumor effector capabilities [22]. mTORC1 inhibition and AMPK activation-induced autophagy possess crucial roles in Treg survival and Treg-mediated immunological tolerance [23]. Autophagy triggered by ULK1 and JNK activation is required for macrophage development at distinct stages. Inhibition of macrophage autophagy stimulates M1-like polarization of TAMs, resulting in enhanced specific immune responses. In contrast, autophagy induced by the binding of IL6 and CCL2 to IL6R and CCR2, respectively, promotes the polarization of macrophages toward the immunosuppressive M2-phenotype TAMs [24,25,26]. The inhibition of p38 MAPK or mTORC1 can prevent the proliferation of neutrophils by activating autophagy [27]. In addition, autophagy can promote the proliferation of myeloid-derived suppressor cells [18].

### Genetic Mutations in Autoimmune and Immune-Mediated Diseases That Impair Autophagy

ATG genes orchestrate and mediate the creation of double-membrane structures that transport intracytoplasmic materials to lysosomes. This mechanism is conserved in all eukaryotic creatures, appears at baseline in almost all cell types, and is stimulated by a variety of internal and extracellular signals. It is necessary for cellular homeostasis, the control of the integrity of cellular proteins and organelles, and organismal adaptability to external stress [28,29]. This lysosomal degradation process is typically defined as requiring 16–20 conserved ATG genes [29]. The core ATG proteins are required but not sufficient for autophagic degradation. Degradation of autophagosomal cargo is impossible without the effective conjugation of the autophagosome with a functional lysosome. In the past ten years, scientists have uncovered a number of components necessary for lysosomal biogenesis, autophagolysosomal fusion, function of lysosomes throughout autophagy, and regeneration of autophagic lysosomes [30,31,32,33]. Recent studies of autoimmune and immune-mediated diseases have identified mutations in a number of genes required for specific autophagy steps.

ATGL16L1 T300A is a prominent CD risk allele. The T300A polymorphism is associated with a caspase 3 cleavage locus that lowers protein amounts. T300A knock-in, hypomorphic, and intestinal knock-out mice display reduced gastrointestinal bacterial elimination, raised cytokine reactions, decreased Paneth cell lysozyme secretion and clearance of IRE1α protein aggregates throughout ER stress, increased enterocyte TNFα-induced necroptosis, imperfections in DC-mediated induction of Tregs, and suppression of mucosal inflammation [34,35]. In IBD, the GPR65 I231L variant of this proton-sensing G protein-coupled receptor influences lysosomal acidity, decreases cellular bacterial elimination, and modifies lipid molecule recycling [36]. IRGM polymorphisms are associated with a higher risk of CD [37]. LRRK2 represents a risk allele for CD by increasing its own kinase activity and reducing autophagic flux, whereas a protective allele enhances autophagic activity [38]. MTMR is a PI3P phosphatase with an autophagy-decreasing effect. In IBD, macrophages from bearers of the risk allele express higher levels of MTMR3 protein and have greater PRR-induced caspase-1 activation and IL-1β secretion [39]. In CD, a mis-sense mutation in CALCOCO2 (NDP52), an autophagy adaptor, diminishes its function and elevates the activation of inflammatory genes by NF-kB [40]. The UC susceptibility gene SMURF1 encodes an E3 ligase involved in mito-, viro-, and xenophagy of intracellular microorganisms [41].

The allele R114W of the ATG16L2 gene is a disease susceptibility gene to SLE [42]. ATG5 polymorphisms are also linked to susceptibility to SLE [43]. Studies on mice show that the process may include ineffective LC3-associated phagocytosis [44]. Intronic mutations of the ATG5 gene are related to systemic sclerosis susceptibility [45]. Many autoimmune disorders, some of them primary immunodeficiency-associated ones, are connected to CLEC16A mutations. Autoimmunity and autophagy deficiencies are related to defective cell mitophagy in Clec16a-deficient mice [46,47,48].

A disease-associated SNP in PTPN2 causes altered autophagosome formation and ineffective bacterial elimination by macrophages and intestinal epithelial cells [49]. PTPN2 mutations alter autophagy in IBD, T1D, and JIA [29,49]. In FMF, disease-associated TRIM20 mutations are incapable of targeting inflammasome components for autophagic degradation [50].

## 3. Autophagy and Carcinogenesis

As a “dual-edged sword” in tumors, autophagy can either stimulate or inhibit tumor growth. The autophagy regulatory network that regulates tumor progression and therapeutic resistance is dependent on cell and tissue types and tumor stages [51].

In general, autophagy inhibits tumor formation by limiting the hazardous buildup of degraded proteins and organelles, specifically mitochondria. By restricting the effects of oxidative stress, persistent tissue injury, and oncogenic signaling, autophagy inhibits tumorigenesis. The essential autophagy gene ATG6/BECN1 encoding the Beclin1 protein has been postulated as a tumor suppressor in breast, ovarian, and prostate malignancies [52]. However, there is no evidence of a Beclin1 mutation or deletion in any other malignancies, indicating that it is still unknown if Beclin1 is a tumor suppressor in the vast majority of human tumors [53]. PARK2, a gene associated with autophagy, has been discovered as a possible tumor suppressor that is commonly lost in human malignancies. Inactivation of PARK2 is responsible for the accumulation of cyclin D and the acceleration of cell cycle progression [54]. Multiple oncogenic pathways are modulated by the signaling adapter p62, including NRF2, mTOR, and NF-κB [55]. Decreased autophagy results in the buildup of p62, which increases hazardous ROS accumulation and chromosomal instability. Autophagy-deficient Kupffer cells boost hepatocarcinogenesis at the initial stage by ROS-triggered inflammation and fibrosis-inducing actions via NF-κB-IL1α/β pathway enhancement [56]. Similarly, ATG gene deletion has been detected in the pancreas, an additional tissue in which prolonged inflammation supports the formation of benign tumors [57]. In addition, the response of death receptor agonists that reduce the proliferation of tumor cells involves autophagy. In the same population of cells, however, excessive autophagy increases Fas-induced apoptosis sensitivity while decreasing TRAIL-induced apoptosis sensitivity [58]. This finding implies that autophagy-induced Fas-mediated apoptosis is cell-type selective. The mechanism is that selective autophagy degrades a negative regulator of Fas-induced apoptosis that is cell type-specific. Hence, the pro-apoptotic effect was limited to a subpopulation of tumor cells [59]. Autophagy can degrade active caspase-8 to inhibit TRAIL apoptotic pathway activation. Hence, autophagy has opposite effects on two very comparable triggers for cell death (FasL and TRAIL), even among identical tumor cells [60].

By destroying EMT inducers, autophagy could facilitate tumor invasion and metastasis formation. Autophagy caused by mTORC inhibition contributes to the considerable decrease in SNAIL and SLUG proteins, hence upregulating cadherin and preventing cancer invasion and metastasis formation [61]. Moreover, DEDD promotes autophagy by interacting directly with PIK3C3 and BECN1, resulting in the autophagy–lysosome-dependent degradation of SNAI and TWIST and a reduction in the process of EMT and metastatic behavior [62].

**Figure 1:**
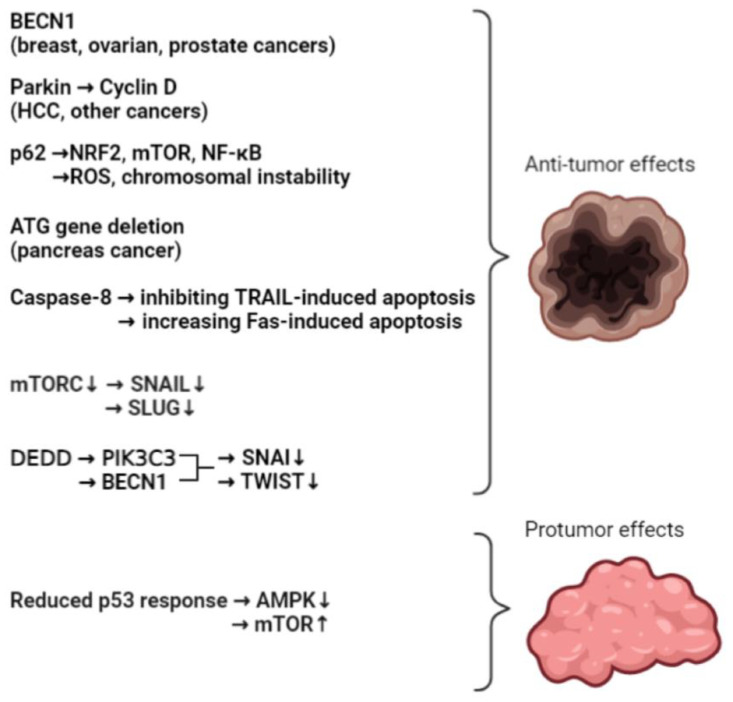
The complex molecular background of the influence of Janus-faced autophagy on tumors. Stated in parentheses are the cancer types in which the feature has been demonstrated. ↓: down-regulation; ↑: up-regulation. The figure was created partly by using BioRender.com, accessed on the 3 April 2023.

Certain malignancies, in contrast, stimulate autophagy and utilize autophagy-mediated recycling to preserve the integrity of mitochondria and energetic homeostasis [63]. Autophagy reduces the p53 signal to enhance cancer growth in stressful situations [64]. The antitumor actions of autophagy blocking in relation to oncogenic Ras in multiple tumor types are p53-dependent, which decreases autophagy by blocking AMPK and activating mTOR, indicating that the absence of tumor suppressor p53 in the presence of oncogenic Ras substantially increases tumor cell proliferation [65,66]. Consequently, autophagy is not defensive in certain conditions and stages of development. It is anticipated that the newly developed p53-stabilizing small molecules will pave the way for future clinical trials by allowing the reactivation of inactive p53 in tumor cells [67] (Figure 1).

### Involvement of Autophagy in Regulation of Cancer Stem Cells and Tumor Microenvironment Function

Autophagy plays an important role in several CSC functions, including maintenance and survival [68,69,70,71,72,73], adaptation to the TME [74,75,76], migration and invasion [77,78], chemo- and immune-resistance [79,80,81,82], and metabolic reprogramming [83,84,85]. Numerous studies suggest that the inflammatory response in the TME is regulated by autophagy via TAMs and CAFs. By influencing monocyte development into macrophages [86,87], monocyte and macrophage recruitment [88,89], and macrophage polarization [90,91], these cells deliver conclusive proof that autophagy functions as a critical regulator of macrophage development. Autophagy also regulates fibroblast development into CAFs; hence, it is the key component of the immune response within the TME [92,93,94].

Interestingly, even in the absence of TME, activation of the TLR9-mediated inflammatory pathway may lead to the appearance of the CSC phenotype. The biological effects of cfDNA depend on its structure and origin [95]. We studied the pathobiological effects of cfDNA administration in HT29 colon adenocarcinoma cells using an in vitro model lacking the TME or the host’s immune system. We found that TLR9 signaling and autophagy have a close relationship which influences the survival of HT29 tumor cells treated with unmodified or manipulated cfDNA [96]. HT29 cells treated with hypermethylated cfDNA also developed colonospheres with high CD133 positivity. By decreasing TLR9 signaling, the anti-proliferative effects of tumorous cfDNA and IGF1R inhibition could be blocked [97]. Autophagy induced by cfDNA and IGF1R inhibition led to the persistence of CD133+ HT29 stem-like cancer cells, which could contribute to CRC recurrence. As HT29 malignant cells are K-Ras wild-type, it cannot be discounted that this phenomenon is partially mediated by the RAS/ERK and PI3K/Akt routes, with a strong connection to pro-inflammatory cytokines IL17, IL22, and IL23 [98].

## 4. Implication of Autophagy in the Commonalities between Autoimmunity and Carcinogenesis

### 4.1. Impact of the Microbiome on Autophagy in Relation to Anticancer Immunity and Self-Tolerance

Microbiome imbalances can lead not only to inflammatory diseases, but also to the growth of tumors (e.g., *Helicobacter pylori* infection and gastric cancer, *Candida* infection and oral tumors) [99,100,101]. In addition to the composition of the microbiome, its activity also influences carcinogenesis. Microbial compounds (e.g., N-nitroso compounds, short-chain fatty acids) can have both direct pro- and anti-tumor effects [102,103]. Microbiome metabolites also influence immune function and, thus, indirectly affect antitumor immunity. Oral *Candida* infection may exert tumor-inhibiting effects by promoting the proliferation of MDSCs [101]. These findings suggest that the microbiome may influence the efficacy of antitumor therapies. This has also been observed, for example, in probiotics containing *Akkermansia muciniphila* or *Bifidobacterium* species during immune checkpoint inhibitor (anti-PD-1) treatments and was found to have beneficial effects [104,105,106].

In some immune-mediated diseases (e.g., CD), the interaction of microbial flora, environmental factors, and the immune systems in genetically pre-disposed individuals underlies the development of the disease [107,108]. In CD, the composition of the gut microbiome is low in diversity, with some strains (e.g., *Firmicutes*) being present in low numbers [109]. TLR4 and IL10 receptor mutations have also been linked to *Mycobacterium avium* subspecies *paratuberculosis* [110]. 

The NOD2/CARD15 gene and its variants are crucial to the development and progression of CD [111,112]. The NOD2 protein encoded by this gene, which has a role in native immunity against bacterial cell walls, is expressed by monocytes, DCs, intestinal epithelial cells, and Paneth cells [112]. Some variants (e.g., L1007fs, G908R, R702W) and polymorphisms (e.g., P268S, IVS8 + 158) of this gene increase the risk of developing CD [110]. In T1D, the structural similarity (i.e., molecular mimicry) among self and microbial antigens (e.g., *Coxsackievirus*, *Rotavirus*) has also been associated with the loss of self-tolerance to pancreatic β-cells [113,114,115].

The complex molecular link among insulin resistance, diabetes, obesity, genomic instability and cancer has been described [116,117]. Punicalgin, a hydrolyzable tannin isolated from pomegranate juice, has been found to ameliorate high-fat diet-induced insulin resistance, hepatic glucose and fatty acid metabolism disturbances, and damage to the liver by inhibiting the IKKβ/NF-κB inflammation route, controlling the intestinal microbiota equilibrium, and enhancing hepatic autophagy activity [118]. These results suggest that manipulation of microbial composition may facilitate the restoration of antitumor immunity or self-tolerance, with important therapeutic implications.

### 4.2. Involvement of Autophagy in Escape from Central Tolerance and Evading Anticancer Immunity

Researchers have long acknowledged the importance of genetic variables in illness onset and distinct subtypes [119]. Diverse alleles of HLA class I and II molecules have been linked to the development of autoimmune disorders (e.g., T1D, celiac disease, MS, RA, etc.) [120,121]. However, the mechanism by which HLA polymorphisms lead to autoimmunity is currently unclear. It is thought that HLA molecules bind autoantigens with varying affinity [122].

Patients suffering from non-muscle-invasive, high-risk bladder cancer have a poor prognosis following routine intravenous BCG therapy. Intracellular BCG infection of cancer cells resulted in a post-transcriptional downregulation of HLA-I membrane expression by inhibiting autophagy flux [123]. After BCG therapy, those with HLA-I-deficient cancer cells exhibited a myeloid immunosuppressive TME with EMT characteristics and poor outcomes. In contrast, following BCG treatment, individuals with HLA-I-competent cancer cells exhibited CD8+ T cell tumor infiltration, an increase in pro-inflammatory mediators, and immune checkpoint-inhibiting compounds. The latter patients had a much better prognosis. It is hypothesized that HLA-I expression in recurrent urinary bladder malignancies following BCG is not the consequence of immunoediting; instead, it originates from an immune subversion mechanism generated directly by BCG on tumor cells and is predictive of a poor prognosis [123].

Other gene polymorphisms (e.g., cytokines, PTPN22, chemokine receptors, inhibitory checkpoint genes, or costimulatory molecules) have also been linked to antigen recognition and immune cell activation [124].

Autoimmunity is characterized by the presence of autoreactive T and B cells that are not eliminated by central tolerance mechanisms [125]. Mutations in the transcriptional AIRE gene are one of the most investigated negative selection defects for T cells. AIRE is predominantly expressed on thymic medullary epithelial cells and serves as the catalyst for tissue-restricted antigen expression. Self-reactive T cells that respond to these antigens are, therefore, killed by negative selection. In the case of AIRE gene defects, autoreactive T cells are discharged into the bloodstream, leading to the development of several autoimmune diseases [126,127]. In humans, mutations in AIRE lead to APS1 [128]. Sex hormones influence the expression of the AIRE gene, causing gender differences in autoimmune disorders [129,130]. Additionally, the AIRE gene has been heavily investigated in relation to reproductive malignancies. In prostate cancer, for instance, AIRE expression contributes to anticancer therapy resistance and enhanced tumor invasion. In AIRE+ prostate tumor cells, IL6 and PGE2 production increases, resulting in the formation of M2-polarized (protumor) TAMs. In addition, the AIRE gene also contributes to prostate tumor progression in a murine graft model [130].

AIRE is also implicated in the regulation of autophagy in THP-1, a human monocytic leukemia cell line. The overexpression of AIRE in THP-1 cells enhanced the quantity of endogenous LC3-II and LC3-expressing vesicles. In addition, the inhibition of autophagy or AIRE knockdown by small interfering RNA mitigated these actions. Contrarily, the expression of p62/SQSTM1 was unaffected by the respective treatment in THP-1 cells [131]. These data suggest that alterations in autophagy caused by the AIRE gene are more likely to contribute to the co-occurrence of autoimmunity and carcinogenesis than is currently known.

LAMPs regulate cellular processes such as phagocytosis, autophagy, lipid transport, and aging, among others. It is hypothesized that LAMP2 is a functional link between autophagy and self-peptide MHC-II processing for selection in cTECs, which in turn influences the development of CD4 T cells and the variety of their TCR arsenal. LAMP2 is a well-known autolysosome maturation mediator. cTECs were discovered to express LAMP2 at a high level. In particular, genetic suppression of Lamp2 in thymic stromal cell exclusively inhibited the generation of CD4 T cells undergoing positive selection [132] without inappropriately directing MHC II-restricted cells to the CD8 lineage. The involvement of LAMP2 in several human cancers (such as CRC, HCC, and adenoid cystic carcinoma, as well as breast, prostate, and lung cancers) has also been described and extensively studied [133,134]. The results presented indicate that the effects of LAMP2-associated alterations of autophagy on CD4 T cells influence antitumor immunity and tumorigenesis [135].

The formation of autoreactive B cells in the periphery is caused by a deficiency in the central tolerance mechanism of B cells. The increased number of autoaggressive B cells is also caused by mutations in PTPN22, BTK, and ADA, as well as impaired rearrangements of the BCR light chain and defects in TLRs [4,136]. Inhibition of PTPN22 influences T and B cell antigen-specific responses, dectin-1 activation in DCs, the formation and function of Tregs, macrophage responses controlled by NOD2, TLRs, and NLRP3, and neutrophil adherence and mast cell stimulation in an IgE-dependent way [137]; these functions make it a target for antitumor therapies. A mis-sense mutation at position 1858 (C3T) is the most investigated single nucleotide polymorphism in the PTPN22 locus, which results in the replacement of an Arg (R) to Trp (W) at position 620. The Lyp620W protein variant (rs2476601) interferes with the negative selection of autoreactive B and T cells in the bone marrow and thymus, respectively. [138]. This protein has been identified in a number of autoimmune conditions, such as T1D, RA, SLE, Graves’ disease, and MG [139,140,141,142]. However, the identical variant of PTPN22 improves the anticancer defense and decreases cancer incidence [137,143]. Holders of the PTPN22(C1858T) mutation possess a reduced risk of skin cancer, whereas homozygotes treated with the anti-PD-L1 antibody atezolizumab have a higher survival rate. In CRC, the majority of important genes were found to be suppressed during tumor formation; however, PTPN22 expression increased at all stages, suggesting its involvement in cancer-related immunity [144]. These data support the notion that immune tolerance is necessary to prevent autoimmune disorders; however, reducing the value for T cell activation may enhance tumor inhibition and the effectiveness of anticancer therapy.

### 4.3. Endocrine Influence on Autophagy, Autoimmunity and Carcinogenesis

Sex hormones may also influence the occurrence of cancer immune evasion and autoimmunity. Immune cells, innate as well as adaptive, produce estrogen α and β receptors (B cells express these receptors at a higher level compared to T cells, NK cells, and monocytes), which are responsible for the activation of tolerogenic actions [145]. Estrogens control T cell differentiation into Th2 and Treg cells; boost the secretion of IL4, IL10, and TGFβ; enhance FoxP3, GATA-3, CTLA-4; and PD-1 expression on T cells, and suppress the Tfh response. [146,147]. During the maturation phase, estrogens modulate B cell tolerance by activating the estrogen receptor. Estradiol is responsible for reducing the generation of B cells while increasing the marginal zone B cell population in the spleen via an increase in BAFF content [148]. Estrogens modulate the TME-infiltrating immune cells. Some of the estrogen receptor mutations enhance the number of tumor-infiltrating Tregs and Th cells [149]. Estrogens have also been shown to affect TAMs by inducing their M2-polarization and, thus, enhancing their suppressive function [150,151].

It has been known for a while that ERRα has a profound effect on host biology, including autophagy [152]. E2 regulates the expression of essential autophagy proteins via various transcription factors, microRNAs, and histone modifications that are downstream of the receptors. The E2-regulated autophagic proteins are implicated in the entire mechanism of autophagy [152,153]. Autophagy is the primary source of cholesterol, which is the precursor for the manufacture of estrogen. E2 in the blood induces negative feedback that reduces hormone levels in circulation. E2 induces NOS3 activation and NO production via membrane ERRs. NO stimulates autophagy by inhibiting the expression of mTOR [153].

### 4.4. Immune Checkpoints, Apoptosis and Autophagy

Immune checkpoints are sensors that transmit inhibiting signs to immune cells, thereby promoting tolerance and avoiding autoimmunity [154]. In mouse models, the absence of CTLA-4, PD-1, BTLA, TIGIT, and VISTA results in massive lymphoproliferation, the development of autoimmune diseases, or fatal multi-organ tissue death [155,156,157,158,159,160]. Polymorphisms in immune checkpoint genes have been linked to the susceptibility of humans to autoimmune disorders [161,162,163,164,165].

CTLA-4 is a fundamental T cell response regulator that is expressed by both Tregs and activated normal T cells. It inhibits antigen presentation and subsequent activation of naïve T lymphocytes by binding competitively to CD80 and CD86 co-stimulatory receptors on APCs [166]. CTLA-4 is responsible for ligand binding and trans-endocytosis-mediated ligand clearance from APCs. Therefore, APCs are transiently deficient in CD80 and CD86 and, as a result, co-stimulation via CD28 is diminished [167]. CTLA-4 is essential for avoiding autoreactivity and its absence in humans leads to immunological dysregulation and PID-associated autoimmunity [168]. In turn, the absence of CTLA-4 causes an increase in CD28 co-stimulation, which results in the development of autoaggressive T cells and Treg dysfunction [168,169]. Expression of CTLA-4 on tumor cells represents a dismal prognosis in melanoma and in pancreatic, nasopharyngeal, and breast cancers [170,171,172,173]. Anti-CTLA-4 antibodies have been found to enhance the anticancer immune response and deplete tumor-infiltrating Tregs by means of antibody-dependent cell-mediated cytotoxicity [174,175]. It has been shown that autophagy suppression contributes to CTLA-4 inhibition resistance in melanoma [176]. CTLA-4 activation suppresses autophagy by limiting LC3B transcription and autophagosome production [177]. Activation of autophagy can restore the expression and suppressive function of CTLA-4; therefore, it could be exploited for a potential curative combination with CTLA-4 inhibitors [176,177].

PD-1 is an additional essential immunological checkpoint for self-tolerance and immune response termination. It promotes apoptosis in activated T lymphocytes when connected to its ligand PD-L1. In experimental models of autoimmunity, blocking PD-1 or PD-L1 has been linked to illness onset and progression [178,179]. PD-L1 is expressed in islet cells of the pancreas, endothelial cells, and placenta, where it protects tissues from autoimmune responses [180]. In T1D, beta cells that produce insulin overexpress PD-L1 in response to an autoimmune onslaught, which corresponds to the degree of CD8+ T cell infiltration [181,182]. The interplay of PD-1 and PD-L1 contributes to the development of inducible Tregs. In vitro and in vivo, PD-L1-negative APCs have a diminished capacity to produce Tregs [183]. Immune checkpoints are critical for the tolerance and, in particular, maintenance of Tregs. These findings are supported by recent reports of autoimmune side effects in cancer patients treated with PD-1/PD-L1 axis antagonists [184,185,186]. In malignancy, effector T cells are continuously exposed to antigen activation in the TME, exhibiting elevated levels of PD-1 for an extended period of time, leading to T cell depletion. This leaves T cells incapable of eliminating tumor cells, resulting in the progression of cancer [187,188]. As a result, cancer cells evade the immune system by using PD-L1, subverting immune surveillance processes that act via PD-L1 expression [189]. The presence of PD-L1 in melanoma cell-derived exosomes further supports a systemic immunosuppressive effect [190], whereby CD4+ [191] and CD8+ [192] T lymphocytes are reduced and cancer cell clearance is impeded.

By boosting mTORC1 signaling and inhibiting mTORC2 signaling, cancer cell-intrinsic PD-L1 can inhibit autophagy. [177]. Due to food depletion, PD-L1/PD-1 interaction can promote autophagy in adjacent T cells [193]. In co-cultures of T cells and tumor cells, a Sigma1 inhibitor induces the degradation of PD-L1 via autophagy and suppresses the functional interaction between PD-1 and PD-L1. Thus, Sigma1 modulators may be able to enhance the tumor immune micro-environment by inhibiting PD-L1/PD-1 [194,195]. It was discovered that inhibiting the PD-L1/PD-1 axis with anti-PD1 or anti-PD-L1 antibodies induces autophagy in cancer cells; therefore, it is an appealing treatment for tumors when combined with autophagy inhibitors [196,197].

Fas is a crucial component of central and peripheral tolerance. FasL binding suppresses excessive immune cell activation on TCR-stimulated lymphocytes (so-called activation-induced cell death) [198]. B cell apoptosis triggered by Fas/FasL is crucial for germinal center responses [198]. FasL inhibits the entrance of activated immune cells to immuno-privileged organs (such as the eye, brain, and placenta) when expressed in these tissues [199]. In humans, mutations in genes associated with the Fas/FasL axis lead to the development of ALPS [198,200]. In ALPS, double-negative, terminally differentiated T cells aggregate and display markers of immune exhaustion [201]. T1D [202], autoimmune thyroid disease [203], and multiple sclerosis [204] have all been linked to an increase in FasL expression. Fas/FasL signaling is characterized by the fact that soluble molecules do not promote apoptosis in contrast to membrane-bound variants [205]. Increased soluble Fas/FasL levels have been demonstrated in SLE and Sjögren’s syndrome [206,207]. In TME, enhanced Fas signaling is primarily caused by MDSC-produced elevated FasL concentrations. This action results in the apoptosis of TILs, which is an important cause of immunotherapy failure [208]. FasL can trigger the death of immune cells in the TME, which is obviously linked to an unfavorable prognosis [208]. Nonetheless, the Fas/FasL system’s significance in cancer growth remains contentious. The ultimate outcome may depend on the level of FasL expression in tumor cells and the concomitant neutrophil-mediated inflammation, according to in vitro studies [209,210]. The Bax/Bcl-2 ratio correlates with the susceptibility of tumors to Fas-mediated apoptosis [211,212,213].

Besides the Fas/FasL system, growing evidence reveals connections between other critical proteins of autophagy and apoptosis, which constitute the fundamental mechanisms underpinning their interplay. Through regulating apoptosis, Bcl-2 family proteins play a crucial role in the etiology of cancer [214]. Bcl-2 transgenic mutations under a B cell-specific immunoglobulin promoter boosted the survival of B and T cells in mice, resulting in splenomegaly, lymphadenopathy, hypergammaglobulinemia, auto-antibody production, and glomerulonephritis [215,216,217]. The relationship between anti-apoptotic protein Bcl-2 and autophagy protein Beclin-1 is essential for modulating the transition between autophagy and apoptosis. Bcl-2 joins to Beclin-1 and dissociates it from class III PI3K, hence inhibiting the autophagic response [218].

Many investigations have reported that ATGs can play a significant role in carcinogenesis by influencing a number of oncogenic and tumor suppressive pathways [219]. In addition to Atg genes, which are involved in the development of autoimmunity in multiple ways, Atg12 has a double role in autophagy and apoptosis, connecting both machineries. Through binding to Bcl-2 via a unique BH3-like motif, unconjugated Atg12 favorably promotes mitochondrial apoptosis [218].

Caspases, a class of cysteine proteases, initiate and regulate apoptotic cascades. A number of malignancies have altered caspase function, with apoptotic evasion designated as the “hallmark of malignancy” [220]. The pyroptosis-related caspases (i.e., Caspase-1, 3, 4, 5, and 11) are intimately implicated in the pathomechanism of several autoimmune illnesses, including SLE, Sjögren’s syndrome, RA, polymyositis, and IBD [221]. Caspase-3 can cleave Beclin-1 to prevent autophagy. Caspase-6 can cleave the autophagy regulators Beclin-1, Atg3, and Atg5. Caspase-9 may increase Atg7-dependent autophagosomal LC3-II production and autophagic function [218].

In inflammation, DNA damage is caused by mitochondrial or nuclear redox imbalance, which initiates the DNA damage response. The signaling protein p53 is implicated in DNA damage response-mediated senescence induction. Increasingly, data suggests that certain organ dysfunctions in autoimmune illnesses are the outcome of inflammation generated by DNA damage-driven senescence [222]. The important tumor suppressor gene TP53 is most commonly altered in human cancers. Mutant p53 frequently acquires novel oncogenic capabilities in addition to losing its tumor suppressive function, a phenomenon known as “gain-of-function.” Increasingly, evidence reveals that p53 mutations are strongly related to advanced cancers and a poor prognosis [223]. In the nucleus, p53 increases the production of pro-apoptotic proteins (e.g., Bax, Bid, PUMA, and Noxa), which initiate the intrinsic apoptotic pathway. In the cytoplasm, p53 increases the expression of the TRAIL and Fas receptors, which initiate the extrinsic apoptotic pathway. p53 is also involved in the control of autophagy. DRAM transcriptional activation promotes autophagy induction and autolysosome formation. DRAM appears to be an integral component of the system that modulates apoptosis and autophagy mediated by p53. Moreover, p53 in the cytoplasm inhibits autophagy by stimulating mTOR signaling. Under conditions of nutritional restriction, p53 inhibits the expression of LC3, which regulates autophagy and safeguards cells against “autophagic burst” [218].

FLIP is an anti-apoptotic protein that suppresses death receptor-mediated apoptosis. Several diseases, including cancer and autoimmune disorders, have been connected to the dysregulation of FLIP expression [224]. FLIP inhibits autophagy by competing with LC3 for Atg3 binding and inhibiting LC3 lipidation [218].

BTLA, TIM-3 and TIGIT are also immunological checkpoints [225,226]. BTLA impacts B cells in addition to decreasing the response in activated T cells [227]. In SLE and MS, B and T cell BTLA expression is diminished [228,229,230]. Decreased BTLA expression on naïve B cells in SLE results in elevated levels of IFNγ and autoantibodies, indicating altered B-cell activation during the disease [228]. In macrophages, BTLA-mediated autophagy and *Mycobacterium tuberculosis* clearance require AKT/mTOR signaling [231]. The expression of BTLA is also present in a wide spectrum of tumor cells. In BTLA-knock-out cells, the overexpression of BTLA reverses the effects of BTLA depletion or HVEM reduction on cell proliferation and colony formation. Upregulation of BTLA or HVEM, in contrast, suppresses cancer cell growth and colony formation [232]. TLR4 activation of monocytes is required for the production of CXCR5^-^PD-1^-^BTLA^-^CD69^high^ tissue-resident IL21+ Tfh cells in the TME of HCC. These Tfh cells stimulate plasma cells, leading to optimal conditions for M2 TAM production and the progress of malignancy [233].

In individuals with autoimmune hepatitis, the number of TIM-3+ T cells declines as IL17 levels rise, which may suggest that TIM-3 deficiency has a hepatotoxic effect. This has been established in mice experiments [234]. The inhibition of intracellular TIM-3 in melanoma cells was observed to enhance MAPK-dependent carcinogenesis [235]. In addition, the MAPK and autophagy pathways collaborate to support the survival of RAS mutant cancer cells [236].

TIGIT is regarded as a hallmark of thymic Tregs with potent suppressive action and stable lineage [237]. TIGIT and CD226 compete with one another to bind to CD155 and its inhibitory CD112 in DCs [237]. The pathogenesis of EAE is influenced by TIGIT–CD226 signaling pathway in T cells. EAE mice lacking CD226 have a beneficial Th17/Treg ratio, increased expression of TIGIT and CTLA-4, enhanced pro-inflammatory cytokine levels, and decreased IL10 production [160,238]. In cancer cells, a novel TIGIT ligand was recently found. Through interactions with CD155 and CD226/TIGIT, artesunate-induced ATG5-related autophagy was observed to improve NK92 cytotoxicity against endometrial tumor cells [239]. It has been demonstrated that Nectin4 binds solely to TIGIT [240]. The TIGIT–Nectin4 interplay reduces NK function, an important component of the anticancer immune response. In addition, antibodies that inhibit Nectin4 have been reported to enhance in vitro and in vivo tumor death [240]. Nectin4 is also capable of sustaining autophagy induction [241].

**Figure 2:**
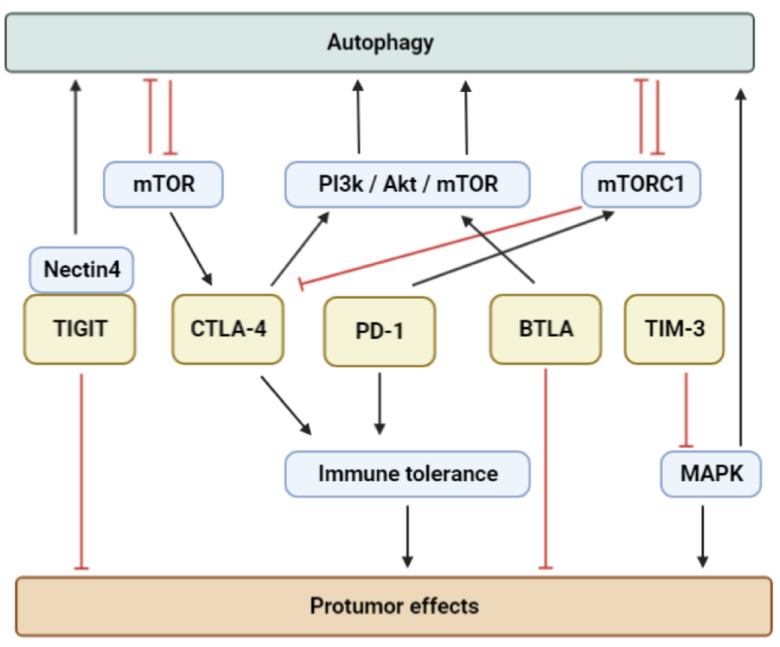
The intricated molecular correlations between immune checkpoints and autophagy in tumor-promoting immune tolerance are depicted in this diagram. The red arrows represent an inhibitory effect, while the black arrows represent a facilitative impact. The figure was created in part by using BioRender.com, accessed on the 3 April 2023.

At multiple stages of cancer development, immunological checkpoint molecules and apoptosis are connected to autoimmunity, while autophagy plays a multidimensional role in this intricate interplay (Figure 2).

### 4.5. Regulatory T and B Cells, Autophagy, Autoimmunity and Carcinogenesis

Thymic Tregs are self-reactive, intermediate-affinity T cell clones that are essential for central tolerance [242,243]. In the peripheral blood, Tregs can be potentially generated from naïve or effector T cells. In addition, certain sub-populations of Tregs can be identified based on the cytokines they release (i.e., type 1 Tregs, type 3 Th cells, and IL35-secreting Tregs) [243]. Functionally, the FoxP3+ population contains Tfr cells that are capable of controlling germinal responses and antibody production [244,245,246].

The primary immunosuppressive mechanisms of Tregs are: (a) strong expression of immune checkpoint inhibitors; (b) tolerance against infections (activation of Tregs by autoantigens results in suppressive activity); (c) secretion of anti-inflammatory cytokines; (d) IL2 deprivation; (e) adenosine accumulation by CD39 and CD73 activity; and (f) immune regulation by extracellular vesicles [247,248,249,250,251].

Tregs display a key function in maintaining self-tolerance as their absence results in IPEX syndrome which, without any treatment, leads to multi-organ autoimmune damage [252,253]. Many autoimmune disorders (e.g., JIA, RA, systemic sclerosis, and SLE) have been linked to quantitative alterations in Tregs [254,255,256,257]. It is challenging to explore the involvement of Tregs in human organ-specific disorders since systemic and local immune responses might vary greatly (e.g., autoimmune thyroid disease, relapsing-remitting MS, T1D, and arthritis) [258,259,260,261,262,263,264]. The transcriptional programs of Th17 and Treg cells are tightly coupled and both are TGFβ-dependent. In response to IL6, Tregs are transformed into Th17 cells, known as IL17+ ex-Tregs. This flexibility decreases suppressive capacity and boosts pro-inflammatory IL-17 and IFNγ production [265,266,267].

Active autophagy in Treg cells promotes their lineage stability and survivability [268]. Foxp3+ Tregs effectively reduce autoimmunity in vivo via CTLA4-dependent regulation of the autophagic processes in DCs [269].

Due to the immunosuppressive cytokine environment and chemotactic factors, Tregs are present in a significant proportion of TMEs. The CCR4 or CCR5 chemokine receptor expression on Tregs permits their migration into the TME [270,271]. The presence of TGFβ in the TME promotes the development of conventional CD4+ T cells into peripheral Tregs [272]. Very active and immunosuppressive Treg cells are present in the TME, in part because they overexpress FoxP3 [273]. Thus, they inhibit antitumor CD8+ T, NK, and NKT cells, as well as M1-polarized macrophages, while promoting DC maturation via IL10, TGFβ, and IDO [274,275,276]. By competitively binding IL2 to conventional T cells and releasing a soluble IL2R component, Tregs remove IL2 and change the actions of cytotoxic T cells. Tregs in the TME augment the expression of PD-1, TIM-3, or LAG-3 via IL35, resulting in the exhaustion of TILs [277,278,279].

The presence of tumor-infiltrating Tregs is associated to a poor prognosis for cancer patients. Substantial intratumoral Treg infiltration suggests that a continuous antitumor T cell response might be finally inhibited [280,281,282]. As autophagy preserves the normal functioning of Tregs by connecting external stimuli and metabolism, the Foxp3CreAtg7^fl/fl^ mouse model was developed to investigate the intrinsic role of autophagy in Tregs and the impact of Treg-restricted autophagy deficiencies on tumor regulation [283,284]. MC38 colon adenocarcinoma cells injected into Foxp3CreAtg7^fl/fl^ mice significantly suppressed tumor growth. At the tumor site, mice consistently displayed high percentages of tumor-infiltrating CD8+ cells, elevated levels of IFNγ expression in effector CD4+ and CD8+ T cells, and a substantial loss of Tregs [283]. These results demonstrate the significance of autophagy in the suppression of immune responses against tumors by Tregs.

The B cell maturation process requires checkpoints (i.e., clonal deletion, receptor editing, and anergy) for tolerance to develop [285,286]. Breg is a subset of B cells that expresses PD-1, TIM-3, and BTLA; it is essential for the maintenance of peripheral tolerance [287,288]. However, no consensus has emerged on the precise definition and phenotype of Bregs [289,290,291,292,293,294,295,296,297,298,299,300,301]. In experimental murine models of autoimmune conditions, modulation of the Breg compartment by adoptive transfer of separated or ex vivo-induced cells has been investigated (i.e., IL10+ Bregs have been shown to reduce inflammation, especially when used in the early stages of the disease) [300,302,303,304,305]. IL10+ B cells and their progenitors are also more frequently found in human autoimmune diseases (e.g., SLE, Sjögren’s syndrome, RA, MS, and autoimmune vesiculobullous skin disease) [292]; however, their role in pathogenesis is still unknown.

CD25^high^CD27^high^CD86^high^CD1^high^IL10^high^TGFβ^high^ human Bregs reduce in a dose-dependent manner the expansion of autologous conventional CD4+ T cells. Moreover, Bregs upregulate the expression of FoxP3 and CTLA-4 in Tregs in a cell-dependent way. When Bregs received prior therapy with TLR9 agonist and CD40L, the effect became even more pronounced [300]. Other research groups have reported that Bregs inhibit DC as well as macrophage production of cytokines and antigen presentation [292,306].

Bregs have a well-defined role in tolerogenesis, producing immunoregulatory cytokines (e.g., IL10 and TGFβ and promoting the contact-dependent repression of autoreactive lymphocytes [307]. However, the mechanisms of the autophagy/Breg interplay in autoimmunity are not well understood.

B cells, including Bregs, can have pro- and anti-tumor actions [308,309,310,311]. It is plausible that tumors and TME transform B-cell infiltrators into tumor-induced Bregs [312,313]. Bregs that infiltrate tumors destroy the CD3 ζ-chain of CD4+ T cells, hence inhibiting T cell proliferation [314]. Bregs produced by tumors promote MDSC development [315] and, in part, contribute to Treg proliferation via IL10 [294,300,316]. TGFβ- or PD-L1-dependent suppression of Th-1 cytokine release and NK cell growth by tumor-induced Bregs in animal models occurs [313]. Moreover, IL35+ Bregs promote tumor growth by transforming T and B cells into cells with regulatory phenotype [317].

Tumor cell-released autophagosomes from mouse cancer cell lines were found to stimulate the development of splenic B cells into CD1d + CD5 + IL10+ Bregs, which could potently suppress CD8+ and CD4+ T cell actions in vitro and in vivo [318].

**Figure 3:**
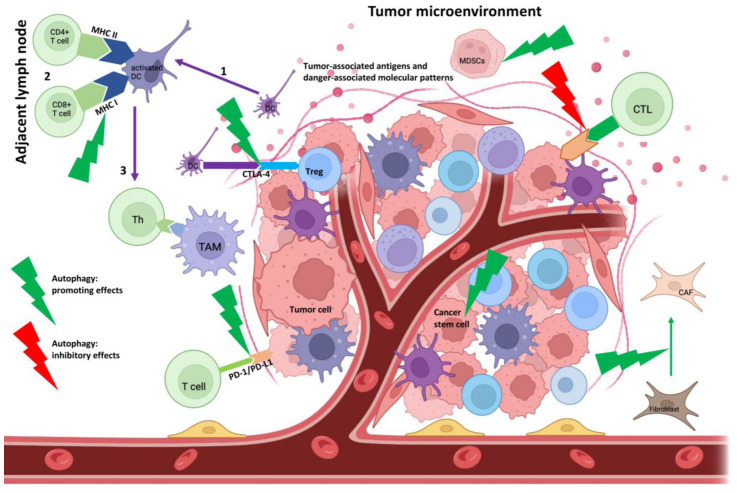
Immunobiological impact of autophagy in TME. Tumor-associated antigens formed in tumor cells and DAMPs from dying tumor cells are taken up by DCs (1). During migration and activation of DCs, antigen presentation to naïve CD4+ and CD8+ T cells occurs through MHC and costimulatory molecules (2). Autophagy can enhance the expression of MHC I molecules. Following antigen recognition, T cells differentiate into CTLs and effector Th cells. The differentiated T cells migrate into TME (3), where autophagy may promote increased PD-L1 and CTLA-4 expression and decreased MHC I expression on malignant cells, thereby inhibiting the effectiveness of cytotoxic T cells. In addition, autophagy promotes the formation and survival of MDSCs and CSCs and may enhance the conversion of fibroblasts into CAFs. Together, these effects may be beneficial for tumor development. The figure was in part created by using BioRender.com, accessed on 4 March 2023.

As previously described, the role of regulatory T and B cells in autoimmunity and carcinogenesis is multiple and complex; autophagy is an integral part of these interactions. This may have important therapeutic implications for the future (Figure 3).

### 4.6. Involvement of γδT Cells in Autophagy, Autoimmunity, and Carcinogenesis

The γδT cells are a CD3+ subset of T lymphocytes that are innate-like and exhibit a TCR composed of γ and δ chains [319]. Besides the peripheral circulation, they reside in the epithelial layers, constituting the majority of IELs [320]. They are involved in sustaining homeostasis and modulating the microbiome, as well as exerting potent anti-inflammatory and antitumor effects [321]. They serve as a link between innate and adaptive immunity, induce rapid immune responses with neutrophils and macrophages against a variety of pathogens, and assist adaptive immune cells in executing their effector functions. In addition, the expression of TCR and NK receptors (e.g., CD94 and NKG2D) enables them to destroy target cells and stimulate additional immune cells [319,320]. 

It is widely recognized that IL17A is essential for the onset and progression of autoimmune conditions [322,323,324]. Innate immune cells, particularly those related to the γδT cell portion, participate in IL17A generation at the initial stage of autoimmune disorders [325]. The γδT cells can be triggered despite the lack of a corresponding TCR ligand, allowing them to function as potent early inflammatory inducers [325]. Several molecules (e.g., IL1, IL6, IL23, TGFβ, or TCR agonists) play a role in the differentiation of the Th17 cytokine signature in vitro [326]. In patients suffering from autoimmune liver conditions (e.g., PBC, PSC, or AIH), there is a significant increase in Vd1+, Vd2+, and Vd3+ γδT cells in the peripheral blood and liver, suggesting the involvement of this particular portion in autoimmunity [327].

In mice, fine particulate matter (with a diameter of <2.5 µm) has been shown to promote lung inflammation and fibrosis by inducing the production of IL17A in γδT and Th17 cells, hence blocking the PI3K/Akt/mTOR-mediated autophagy machinery in the bronchial epithelium [328]. MAPK family inactivation was found to block keratinocyte autophagy, which is associated with the worsening of psoriasis in human and mouse models. Krt14Cre/+-hmgb1f/f mice exhibited lessened psoriatic inflammation as a result of the crucial interaction between γδT cells and keratinocyte-specific HMGB1-associated autosecretion [329]. Further investigation of the role of γδT cells in autophagy and autoimmunity is warranted as they could provide an ideal basis for newly developed cell-based therapies with immunomodulatory effects. 

The γδT cells are a component of the TME and are known to influence the antitumor immune response to a wide variety of malignancies [330]. Targeting immune checkpoints has been shown to restore the malfunctioning condition of γδT cells in the TME. Moreover, immune checkpoint inhibition induces antitumor effects by enhancing the proliferation, activation, and cytotoxic effect of γδT cells [331,332]. It has been shown that isolated human γδT cells induce autophagy in myeloma cells by suppressing the expression of autophagy-related phospho-AKT, PI3K, and phospho-mTOR, while increasing the expression of BECN1 and AMPK [333]. On the other hand, a previous study highlighted the role of IL17A-producing γδTILs in promoting tumor growth in CRC patients; however, these findings were not corroborated by subsequent research [334,335].

As can be seen, the rapid activation and cytotoxic nature of γδT cells make them promising candidates for use in cell-based immunotherapies; however, under certain conditions they may induce pro-tumor functions. Investigation of the tumor-influencing role of γδT cells via autophagy is certainly warranted and could form the basis for novel, individualized treatments.

### 4.7. Myeloid-Derived Suppressor Cells and Macrophages in Relation to Autophagy, Autoimmunity and Carcinogenesis

MDSCs are found in malignancies as active, immature myeloid cells with immunosuppressive functions. They are classified into two distinct populations: M-MDSCs and PMN-MDSCs [336]. Many cytokines (e.g., GM-CSF, VEGF, SCF, prostaglandins, TNFα, IFNγ, and IL18) have a role in their differentiation and, hence, the production of immunosuppressive TME and maintenance of CSCs [337]. MDSCs suppresses the activity of TILs, EMT, and angiogenesis, as well as the development of the pre-metastatic niche [338,339]. Additionally, their iNOS activity increases NO generation, which results in T cell death and suppressed proliferation, as well as inhibited antigen presentation in DCs [340,341]. They inhibit T cell growth, survival, and TCR signaling via their elevated ROS levels [342,343,344]. By arginase I overexpression, they deplete the TME of critical amino acids, which impairs T cell activation and proliferation [345,346]. Moreover, they inhibit T cells by downregulating the CD3 ζ-chain of the TCR complex [347]. By activating IDO, they inhibit the proliferative capacity and survival of T cells and stimulate Treg activation [348,349,350]. By generating peroxynitrite, these cells nitrate the TCR complex and render cytotoxic T lymphocytes unresponsive to certain antigens presented by MDSCs [351,352,353]. MDSCs are the major source of the immunosuppressive adenosine [354,355], hence suppressing T cell activation and effects, predominantly via A2A and A3 adenosine receptors [356]. MDSC-produced IL10 and TGFβ enhance the T cell-to-Treg transition and inhibit the activation of T and NK cells, as well as DC activity [357,358]. Via TGFβ synthesis, they stimulate EMT in cancer cells [339,359] and create pro-tumorigenic M2 macrophages and N2 neutrophils [339,359,360,361].

The persistence of TME cells, such as Tregs and MDSCs, is strictly correlated with HMGB1-induced autophagy and tumor resistance to immune surveillance [362]. Autophagy specifically modifies the metabolism, longevity, and development of MDSCs, being essential for MDSC regulation. Autophagy displays a significant effect on MDSC modulation by inhibiting antitumor immunity, whereas inhibiting autophagy restricts tumor growth and increases immunity against malignant cells [363].

The immunosuppressive capacity of MDSCs has also raised attention regarding their function in autoimmune disorders. MDSCs have also been split into two subgroups in immunological studies: M-MDSCs and PMN-MDSCs [364,365]. In an EAE animal model, arginase-I-producing MDSCs were identified in the spinal cord, which showed tropism to demyelinated areas of the CNS; their numbers correlated with the number of apoptotic T cells, disease progression, and clinical status, particularly at the end of the active phase [365,366]. In humans with relapsing-remitting multiple sclerosis, the number of PMN-MDSCs in the circulation during relapse were considerably higher than during remission or in the control group. Based on in vitro tests, it has been demonstrated that PMN-MDSCs suppress the growth of autologous T cells, suggesting a role in the induction of remission [367].

Numerous forms of autoimmune disorders are associated with elevated levels of MDSCs, such as T1D, RA, SLE, IBD, and autoimmune hepatitis [368]. The increasing frequency of MDSCs in individuals with RA and SLE is related to disease progression [369,370]. The ratio of Th17 cells to MDSCs was discovered to be inversely proportional in RA [371]. Th17 cells were initially identified as a cause of some inflammatory disorders. Furthermore, MDSCs have been shown to play a pro-inflammatory function and can trigger arthritis with the help of Th17 cells [369]. The concept of harnessing the suppressive activity of MDSCs for therapeutic purposes inspired experiments involving MDSC adoptive transfer into diabetes-prone mice; these transfers effectively avoided the development of autoimmune diabetes and induced tolerance to the mice’s own antigens through the induction of Tregs [372].

Within infection-related inflammatory circumstances, it was shown that inhibiting autophagy contributes to the aggregation and inhibitory effect of PMN-MDSCs by encouraging the activation of STAT3 signals, indicating that autophagy could have a crucial role in controlling the aggregation and function of MDSCs [373]. Moreover, in tuberculosis, MDSCs were found to express TLR4, mTOR, and IL6 as compared to healthy controls. HMBG1 serum concentration was in parallel to mTOR during treatment [374]. These findings suggest that MDSCs have a close relationship with the complex process of autophagy in certain infectious inflammations. Since infections can trigger autoimmune reactions, the possibility of MDSC/autophagy interaction in autoimmune diseases cannot be excluded; however, no specific proof has been presented.

Macrophages, a class of highly diverse immune cells, are capable of polarizing to various phenotypes in response to the microenvironment. M1-polarization refers to conventionally activated, pro-inflammatory macrophages, whereas M2-polarization refers to alternatively activated, anti-inflammatory macrophages [375].

The recruitment of TAMs to the TME is driven by chemokines (e.g., CCL2) in lung and breast cancers, as well as glioblastoma [376,377,378,379]. In addition, via CCL2 production, TAMs recruit even more macrophages and enhance their M2-polarization [380,381]. TAMs are capable of producing IL8, which has been linked to a dismal prognosis regardless of the amount of CD8+ T cells in the tumor [382]. TAMs can also generate IL6, IL10, and TGFβ. IL6 in conjunction with IL6R can activate anti-apoptotic pathways and extend tumor cell survival [375]. Additionally, TAMs release inflammatory mediators, such as PGE2 and MMP-7, thereby inhibiting DC and macrophage activation caused by TLR or IFNγ. Moreover, they indirectly impede T cell identification of tumor antigens by directly inducing genes that inhibit APC function [383].

Despite the fact that macrophage-mediated phagocytosis plays a crucial role in immune surveillance in malignancies, it has been shown that phagocytosis is significantly compromised, a phenomenon that is primarily due to the autophagy machinery in tumor cells [384,385]. Moreover, autophagy enhances MHC-II expression in macrophages and MHC-I expression in tumor cells [385].

A T1D animal model has proven the permanent existence and function of macrophages as APCs in peripheral tissues [386]. In numerous autoimmune diseases (e.g., MS, RA, SLE, SSc), M1/M2 macrophage subgroup imbalances have been identified [387,388]. Recent investigations utilizing multiparametric analysis on the pancreas of T1D patients have shown the existence of macrophages with mixed M1/M2 features, indicating the remarkable adaptability of these cells [389,390]. According to studies, the polarization of macrophages in EAE follows the normal trend of the disease [391]. In a murine model of SLE, adoptive transfer of M2-polarized macrophages ameliorated the disease [392] and, in NOD mice, delayed the onset of diabetes by targeting the site of insulitis [393].

SQSTM1/p62-mediated clearance of damaged mitochondria regulates the activation of NLRP3-inflammasome in macrophages; deletion of SQSTM1/p62 generates enhanced inflammasome activation and overproduction of IL-1β [394]. Notwithstanding the fact that this process inhibits excessive IL1β-dependent inflammation, other reports have demonstrated that autophagy increases NF-kB action in certain tissue macrophages [395].

MDSCs and macrophages display a crucial role in both autoimmunity and carcinogenesis; the complex process of autophagy is involved at several points in their development, function, and regulation of other immune system elements. This will not only aid the understanding of the pathogenesis of these disorders, but also identify new therapeutic targets in the future (Figure 3).

### 4.8. The Cross-Talk of Cell-to-Cell Interactions with Autophagy, Autoimmunity, and Carcinogenesis

Emperipolesis, entosis, and cell cannibalism have been described as cell engulfment phenomena that can even involve tumor cells [396,397]. Emperipolesis is the phagocytosis of the host’s intact hematopoietic cells (mainly neutrophils, lymphocytes, and plasma cells) by other, sometimes tumorous cells. Entosis represents a mechanism of homogenous live-cell invasion, such that the invading cell seems to take the initiative in being internalized. Cannibalism is the active internalization and destruction of dead or living tumor cells by other engulfing cells [396]. In recent years, a growing body of evidence suggests that these cell-to-cell phenomena are linked to autophagy by multiple strands [398]. 

Emperipolesis is a characteristic feature of AIH, which is linked to severe necrotizing inflammation and advanced fibrosis [399]. Emperipolesis is frequently described in conjunction with interface hepatitis, hepatocyte rosettes, and plasma cell infiltration. The vast majority of lymphocytes engulfed by phagocytosis in liver cells are CD8+ T cells and are mainly observed in the area of caspase-3-induced apoptosis [399]. NK cells are involved in liver fibrosis [400]. HSCs impair the killing effect of NK cells by TGFβ secretion [401]. NK cells can initiate mitophagy, a selective form of autophagy, after virus infections, hence maintaining their own survival [402]. Autophagy is also involved in the transition of effector NK cells to long-live memory cells, partly via an ATG3-dependent mechanism [402]. Moreover, ATG5 deletion interrupts the development and death of NK cells by causing severe mitochondrial damage and ROS generation [403]. To accelerate development and maturation, the collaboration between ATG7 and FoxO1 triggers the cytosolic autophagy flux of immature NK cells [403].

Recent research has shown that overexpression of p62 is linked to lymphocytic emperipolesis and cytoplasmic vacuoles with eosinophilic inclusions in fumarate hydratase-deficient RCC, which is a rare form of renal cancer. The upregulation of p62 is considered to be evidence of defective autophagy [404] and cannot be excluded as a major contributor to the aggressive nature of this tumor type.

It has been demonstrated that the autophagy process contributes to the demise of engulfed cells. Entotic vacuole membranes surrounding engulfed cells recruit LC3 via autophagy machinery-related proteins (e.g., ATG5, ATG7, and VPS34) [405,406]. Internalized cells display some characteristics of autophagic cell death, including an overabundance of autophagosomes and an elevated level of autophagy flux [405]. It is hypothesized that in liver cells, pro-inflammatory cytokines, such as TNFα, IFNγ, and IL6, may inhibit T cell release following entosis [407]. This would explain why CD4+ T cells have a tolerogenic mechanism in the liver to prevent further inflammation. Although the majority of pro-inflammatory cytokine treatments did not affect the percentage of T cells released from hepatomas, shorter incubation durations with stimuli resulted in a greater percentage of T cells released from HepG2-CD81s than 24-h incubations. This result indicates that the organization of hepatomas may affect the rate and frequency of entosis and release. It is crucial to acquire a deeper understanding of the causes of entosis and the discharge of CD4+ T cells; this allows T cells to be manipulated therapeutically to prevent the development of inflammatory liver diseases [407].

It is theorized that entotic cell death serves as a tumor suppressor by promoting the demise of entotic tumor cells [408]. Nevertheless, entosis can result in polyploidy and aneuploidy, which support the progression of tumors. In addition, multinucleation is frequently observed in incorporating host cells after glucose starvation, mitosis, and matrix deadhesion [408,409]. Entosis sometimes does not result in the death of the entotic cell. Consequently, under certain conditions, entotic inner cells retain viability, multiply inside the exterior cell, and eventually leave the outer cell [408].

The interpretation of entosis as either pro- or anti-tumorigenic is not entirely evident at this time; however, the majority of authors believe that it confers a survival advantage to the engulfing cell [410]. The majority of published clinico-histopathological studies of entosis link its presence to a more aggressive phenotype and a poorer prognosis. High numbers of entosis were found to be positively associated with improved survival for anal cancer and certain subtypes of breast cancer, whereas low numbers had a favorable prognostic value for rectal and bronchial malignancies, HNSCC, and PDAC [410]. Investigating the relationship between entosis and autophagy could lead to the development of new anticancer therapeutic targets. 

Immature T cells internalized by TNCs in an MHC-driven manner differentiate into αβTCR^high^CD4 + CD8 + CD69+ cells and subsequently exit TNCs [411,412]. TNCs have a negative selection role in addition to a positive selection function that promotes TCR transformation. Indeed, they select anergic thymocytes for removal at an early stage of intracellular death processes. Recently, it has been shown that concomitant expression of K8- and K5-cytokeratins, P63, TRA, and AIRE in TNCs contribute to negative selection through the expression of autoantigens [413].

In the case of nutrient depletion, mTORC1 activity decreases, which induces autophagy and macropinocytosis and supports the metabolism of tumor cells [414]. The mTORC1-regulated ULK kinase complex that promotes autophagy is implicated in this process [415]. TM9SF4 positively modulates autophagy and cannibalism and contributes to inhibiting mTORC1 activity in response to nutrient deprivation [416]. Elucidating the association between cannibalism and autophagy may help to improve understanding of the pathomechanism of immune-mediated diseases and cancer, as well as the development of new types of targeted therapies. 

## 5. Future Perspectives

Understanding immune tolerance in the TME and the underlying mechanisms of autoimmunity may aid in the generation of an effective antitumor immune response and disrupt cancer immune tolerance. A deeper insight into the pro- and anti-tumor and pro- and anti-inflammatory effects of autophagy on immune function at multiple levels may also provide a theoretical and practical basis for new therapies for tumor diseases and autoimmune pathologies. We believe that integrating knowledge gained from research on autoimmune diseases, carcinogenesis, and autophagy could lead to significant advances in each of these areas.

Autophagy activators, such as rapamycin, resveratrol, retinoic acid, everolimus, spermidine, and vitamin D [417,418,419,420,421,422,423,424,425,426], and autophagy blockers such as chloroquine [427,428], have been utilized for the management of a variety of immune-mediated disorders. Additionally, it has been discovered that certain anti-autoimmune agents are connected with autophagy. Glucocorticoids, an anti-inflammatory medication, have been discovered to activate autophagy [429].

Therapies, acting in part through the influence of autophagy, can be used not only in the treatment of autoimmune diseases but also in cancer therapy [430,431,432,433,434,435,436]. Existing therapeutic strategies are already reaping the benefits of the knowledge exchange between the two research fields. The anticancer action of CAR T cell therapies is partly carried out through the manipulation of autophagy [437,438]. In addition, the treatment options of CAR Tregs in autoimmune diseases are also being investigated [439] as antigen-specific Tregs have been shown to control autoaggressive immune cells more effectively than polyclonal regulatory T cells [440].

An additional instance of a treatment strategy involving autophagy in tumorous and autoimmune disorders is adoptive cell therapy. Restoring autophagy flux enhances the effectiveness of TIL-based adoptive cell therapy [441]. It was revealed that miR-142-3p inhibits the mRNA and protein expression of ATG16L1, which has been associated with autoimmunity. In contrast, miR-142-3p knockdown increased the proliferative capacity, survival, and action of thymic Treg cells in vitro and in vivo. This study revealed a novel approach for boosting the efficacy of thymic Treg cells and the autophagy process by raising ATG16L1 mRNA and protein [442].

The manipulation of autophagy in relation to adoptive cell transfer has shown promise in several autoimmune disease models [443,444]. Immune checkpoint inhibitors, which also affect autophagy, have been shown to be successful in cancer therapy [176] and the treatment of autoimmune diseases [445].

Nonetheless, the safety and risk of immune checkpoint inhibitors in patients suffering from autoimmune diseases has been a major concern due to the immunological activations that these medicines induce. Numerous retrospective studies have begun to investigate this subject and have discovered that autoimmunity is frequently increased by immune checkpoint inhibitor medication; however, it is routinely managed with standard care algorithms and multidisciplinary surveillance [445].

## 6. Conclusions

The purpose of this review was to provide an explanation for the diverse range of processes that regulate autoimmunity and immune tolerance to cancer, as well as their complex and multifaceted relationship with autophagy.

Protective advantages of autophagy modulators on autoimmune disease models have been reported, demonstrating their growing importance in the management of immune-mediated disorders. Recent investigations have examined not only the intrinsic autophagy activities within tumor cells, but also the participation of autophagy in the TME as well as related immune cells. In addition, other autophagy-related mechanisms different from classical autophagy that utilize autophagic machinery components and may contribute to malignant disease have been discovered. Increasing evidence on the influence of autophagy and related processes on cancer development and progression has guided efforts to develop antitumor therapies based on autophagy suppression or stimulation.

## Data Availability

No new data were created.

## Abbreviations

ADA: adenosine deaminase; AIH: autoimmune hepatitis; AIRE: autoimmune regulator gene; AKT: Ak strain transforming, a serine/threonine protein kinase; ALPS: autoimmune lymphoproliferative syndrome; AMPK: AMP-activated protein kinase; APCs: antigen presenting cells; APS1: autoimmune poly-endocrinopathy syndrome type 1; ATG16L1: autophagy related 16 like 1; Atg7: autophagy-related protein 7; ATG8: autophagy-related protein 8; BAFF: B-cell activating factor; Bax: Bcl-2-associated X protein; BCG: Bacillus Calmette–Guérin; Bcl2: B-cell lymphoma 2; BCR: B-cell receptor; BECN1: Beclin 1; BH3: Bcl-2 homology 3; Bid: Bcl-2 interacting domain; Breg: regulatory B cell; BTK: bruton tyrosine kinase; BTLA: B and T lymphocyte attenuator; CAFs: cancer-associated fibroblasts; CALCOCO2 (NDP52): calcium-binding and coiled-coil domain-containing protein 2; CAR T: chimeric antigen receptor T cell; CARD15: caspase recruitment domain-containing protein 15; CCL: chemokine ligand; CCR: chemokine receptor; CD: Crohn’s disease; cfDNA: cell-free DNA; CLEC16A: C-type lectin domain family 16; CRC: colorectal cancer; CSC: cancer stem cell; cTECs: cortical thymic epithelial cells; CTLA-4: cytotoxic T-lymphocyte antigen-4; CTLs: cytotoxic T lymphocytes; DAMP: danger-associated molecular pattern; DC: dendritic cells; DEDD: DNA-binding protein; DNA: deoxyribonucleic acid; DRAM: damage-regulated autophagy modulator; E2: 17β-estradiol; EAE: experimental autoimmune encephalomyelitis; EMT: epithelial-to-mesenchymal transition; ER: endoplasmic reticulum; ERK: extracellular signal-regulated kinase; ERRα: estrogen-related receptor alpha; ERRs: estrogen-related receptors; Fas: CD95; FasL: CD95 ligand; FLIP: caspase 8 (FLICE)-like inhibitory protein; FMF: familial Mediterranean fever; FoxO1: forkhead box protein O1; FoxP3: forkhead box P3; GATA-3: GATA binding protein 3; GM-CSF: granulocyte-macrophage colony-stimulating factor; GPR65: proton-sensing G protein-coupled receptor; HCC: hepatocellular carcinoma; HLA: human leukocyte antigens; HMGB1: high mobility group box 1; HNSCC: head and neck squamous cell carcinoma; HSCs: hepatic stellate cells; HVEM: herpesvirus entry mediator, a BTLA ligand; IBD: inflammatory bowel diseases; IDO: indoleamine 2,3-dioxygenase; IEL: intraepithelial lymphocyte; IFNγ: interferon gamma; IgE: immunoglobulin E; IGF1R: insulin-like growth factor 1 receptor; IgG: immunoglobulin G; IgM: immunoglobulin M; IKKβ: inhibitor of nuclear factor kappa-B kinase subunit beta; IL: interleukin; IL6R: interleukin 6 receptor; iNOS: inducible nitric oxide synthase; IPEX: immune dysregulation, poly-endocrinopathy, enteropathy, and X-linked syndrome; IRE1α: inositol-requiring transmembrane kinase endoribonuclease-1 alpha; IRGM: immunity-related GTPase family M protein; ITIM: immunoreceptor tyrosine-based inhibitory motif; JIA: juvenile arthritis; JNK: c-Jun N-terminal kinase; LAG-3: lymphocyte-activation gene 3; LAMP: lysosomal-associated membrane proteins; LC3: microtubule-associated protein 1A/1B-light chain 3; LRRK2: leucine-rich repeat kinase 2; MAPK: mitogen-activated protein kinase; MDSCs: myeloid-derived suppressor cells; MG: myasthenia gravis; MHC: major histocompatibility complex; M-MDSCs: mononuclear MDSCs; MS: multiple sclerosis; MTMR: myotubularin-related proteins, PI3P phosphatase; mTOR: mammalian target of rapamycin; mTORC1: mammalian target of rapamycin complex 1; MyD88: myeloid differentiation primary response 88; NF-kB: nuclear factor kappa B; NK: natural killer cell; NKT: natural killer T; NLRP3: nucleotide-binding domain, leucine-rich-containing family, pyrin domain-containing-3; NLRs: NOD-like receptors; NO: nitric oxide; NOD mice: non-obese diabetic mice; NOD2: nucleotide-binding oligomerization domain-containing protein 2; NOS3: nitric oxide synthase 3; Noxa: phorbol-12-myristate-13-acetate-induced protein 1; NRF2: nuclear factor erythroid 2–related factor 2; p38MAPK: p38 mitogen-activated protein kinases; PARK2: Parkin; PBC: primary biliary cholangitis; PD-1: programmed cell death receptor 1; PDAC: pancreatic ductal adenocarcinoma; PD-L1: programmed death-ligand 1; PGE2: prostaglandin E2; PID: primary immunodeficiency; PI3P: phosphatidylinositol-3-phosphate; PIK3C3: phosphatidylinositol 3-kinase catalytic subunit type 3; PMN-MDSCs: polymorphonuclear/granulocytic MDSCs; PRRs: pattern recognition receptors; PSC: primary sclerosing cholangitis; PTPN2: protein tyrosine phosphatase non-receptor type 2; PUMA: p53 upregulated modulator of apoptosis; RA: rheumatoid arthritis; RAS: Rat sarcoma virus; RCC: renal cell carcinoma; RIP1: receptor-interacting serine/threonine–protein kinase 1; RNA: ribonucleic acid; ROS: reactive oxygen species; SCF: stem cell factor; SLE: systemic lupus erythematosus; SLUG: SNAI2, a zinc-finger transcription factor; SMURF1: Smad ubiquitination regulatory factor-1; SNAI: SNAIL, a zinc finger protein; SNAIL: zinc finger protein SNAI1; SNP: single nucleotide polymorphism; SQSTM1: sequestosome-1; STAT3: signal transducer and activator of transcription 3; T1D: type 1 diabetes; TAM: tumor-associated macrophage; TCR: T cell receptor; Tfh: follicular helper T cell; Tfr: follicular regulatory T cell; TGFβ: transforming growth factor-beta; Th: helper T cell; Th2: type 2 helper T cell; TIGIT: T-cell immunoreceptor with immunoglobulin and ITIM domain; TILs: tumor-infiltrating lymphocytes; TIM-3: T-cell immunoglobulin and mucin domain-3; TLRs: toll-like receptors; TM9SF4: transmembrane 9 superfamily member 4; TME: tumor microenvironment; TNC: thymic nurse cell; TNFα: tumor necrosis factor alpha; TP53: tumor protein 53; TRA: tissue-restricted antigen; TRAIL: TNF-related apoptosis inducing ligand; Treg: regulatory T cell; TRIF: TIR-domain-containing adapter-inducing interferon-β; TRIM20: tripartite motif 20; TWIST: Twist-related protein; UC: ulcerative colitis; ULK1: unc-like autophagy activating kinase 1; VEGF: vascular endothelial growth factor; VISTA: v-domain Ig suppressor of T-cell activation; VPS34: vacuolar protein sorting 34.

## References

[1] ElTanbouly M.A., Noelle R.J. (2021). Rethinking peripheral T cell tolerance: Checkpoints across a T cell’s journey. Nat. Rev. Immunol..

[2] Hanahan D., Weinberg R.A. (2011). Hallmarks of cancer: The next generation. Cell.

[3] Bates J.P., Derakhshandeh R., Jones L., Webb T.J. (2018). Mechanisms of immune evasion in breast cancer. BMC Cancer.

[4] Sakowska J., Arcimowicz Ł., Jankowiak M., Papak I., Markiewicz A., Dziubek K., Kurkowiak M., Kote S., Kaźmierczak-Siedlecka K., Połom K. (2022). Autoimmunity and Cancer-Two Sides of the Same Coin. Front. Immunol..

[5] Doria A., Gatto M., Punzi L. (2013). Autophagy in human health and disease. N. Engl. J. Med..

[6] Levine B., Mizushima N., Virgin H.W. (2011). Autophagy in immunity and inflammation. Nature.

[7] Yin H., Wu H., Chen Y., Zhang J., Zheng M., Chen G., Li L., Lu Q. (2018). The Therapeutic and Pathogenic Role of Autophagy in Autoimmune Diseases. Front. Immunol..

[8] White E. (2012). Deconvoluting the context-dependent role for autophagy in cancer. Nat. Rev. Cancer.

[9] Rao S., Yang H., Penninger J.M., Kroemer G. (2014). Autophagy in non-small cell lung carcinogenesis: A positive regulator of antitumor immunosurveillance. Autophagy.

[10] Amaravadi R., Kimmelman A.C., White E. (2016). Recent insights into the function of autophagy in cancer. Genes Dev..

[11] Yang A., Herter-Sprie G., Zhang H., Lin E.Y., Biancur D., Wang X., Deng J., Hai J., Yang S., Wong K.K. (2018). Autophagy Sustains Pancreatic Cancer Growth through Both Cell-Autonomous and Nonautonomous Mechanisms. Cancer Discov..

[12] Galluzzi L., Green D.R. (2019). Autophagy-Independent Functions of the Autophagy Machinery. Cell.

[13] Levy M.J.M., Thorburn A. (2020). Autophagy in cancer: Moving from understanding mechanism to improving therapy responses in patients. Cell Death Differ..

[14] Gao P., Liu H., Huang H., Sun Y., Jia B., Hou B., Zhou X., Strober W., Zhang F. (2022). The Crohn Disease-associated ATG16L1T300A polymorphism regulates inflammatory responses by modulating TLR- and NLR-mediated signaling. Autophagy.

[15] Xu Y., Jagannath C., Liu X.D., Sharafkhaneh A., Kolodziejska K.E., Eissa N.T. (2007). Toll-like receptor 4 is a sensor for autophagy associated with innate immunity. Immunity.

[16] Delgado M.A., Elmaoued R.A., Davis A.S., Kyei G., Deretic V. (2008). Toll-like receptors control autophagy. EMBO J..

[17] Pan H., Chen L., Xu Y., Han W., Lou F., Fei W., Liu S., Jing Z., Sui X. (2016). Autophagy-associated immune responses and cancer immunotherapy. Oncotarget.

[18] Janji B., Berchem G., Chouaib S. (2018). Targeting autophagy in the tumor microenvironment: New challenges and opportunities for regulating tumor immunity. Front. Immunol..

[19] Műzes G., Sipos F. (2012). Anti-tumor immunity, autophagy and chemotherapy. World J. Gastroenterol..

[20] Zhong Z., Sanchez-Lopez E., Karin M. (2016). Autophagy, Inflammation, and Immunity: A Troika Governing Cancer and Its Treatment. Cell.

[21] Arnold J., Murera D., Arbogast F., Fauny J.D., Muller S., Gros F. (2016). Autophagy is dispensable for B-cell development but essential for humoral autoimmune responses. Cell Death Differ..

[22] Wang S., Xia P., Huang G., Zhu P., Liu J., Ye B., Du Y., Fan Z. (2016). FoxO1-mediated autophagy is required for NK cell development and innate immunity. Nat. Commun..

[23] Zeng H., Yang K., Cloer C., Neale G., Vogel P., Chi H. (2013). mTORC1 couples immune signals and metabolic programming to establish T(reg)-cell function. Nature.

[24] Jacquel A., Obba S., Solary E., Auberger P. (2012). Proper macrophagic differentiation requires both autophagy and caspase activation. Autophagy.

[25] Zhang Y., Morgan M.J., Chen K., Choksi S., Liu Z.G. (2012). Induction of autophagy is essential for monocyte-macrophage differentiation. Blood.

[26] Chen P., Cescon M., Bonaldo P. (2014). Autophagy-mediated regulation of macrophages and its applications for cancer. Autophagy.

[27] Rožman S., Yousefi S., Oberson K., Kaufmann T., Benarafa C., Simon H.U. (2015). The generation of neutrophils in the bone marrow is controlled by autophagy. Cell Death Differ..

[28] Levine B., Kroemer G. (2008). Autophagy in the pathogenesis of disease. Cell.

[29] Levine B., Kroemer G. (2019). Biological Functions of Autophagy Genes: A Disease Perspective. Cell.

[30] Settembre C., Fraldi A., Medina D.L., Ballabio A. (2013). Signals from the lysosome: A control centre for cellular clearance and energy metabolism. Nat. Rev. Mol. Cell Biol..

[31] Yu L., Chen Y., Tooze S.A. (2018). Autophagy pathway: Cellular and molecular mechanisms. Autophagy.

[32] Shen H.M., Mizushima N. (2014). At the end of the autophagic road: An emerging understanding of lysosomal functions in autophagy. Trends Biochem Sci..

[33] Chen Y., Yu L. (2017). Recent progress in autophagic lysosome reformation. Traffic.

[34] Tschurtschenthaler M., Adolph T.E., Ashcroft J.W., Niederreiter L., Bharti R., Saveljeva S., Bhattacharyya J., Flak M.B., Shih D.Q., Fuhler G.M. (2017). Defective ATG16L1-mediated removal of IRE1α drives Crohn’s disease-like ileitis. J. Exp. Med..

[35] Matsuzawa-Ishimoto Y., Shono Y., Gomez L.E., Hubbard-Lucey V.M., Cammer M., Neil J., Dewan M.Z., Lieberman S.R., Lazrak A., Marinis J.M. (2017). Autophagy protein ATG16L1 prevents necroptosis in the intestinal epithelium. J. Exp. Med..

[36] Lassen K.G., McKenzie C.I., Mari M., Murano T., Begun J., Baxt L.A., Goel G., Villablanca E., Kuo S.-Y., Huang H. (2016). Genetic Coding Variant in GPR65 Alters Lysosomal pH and Links Lysosomal Dysfunction with Colitis Risk. Immunity.

[37] Chauhan S., Mandell M.A., Deretic V. (2016). Mechanism of action of the tuberculosis and Crohn disease risk factor IRGM in autophagy. Autophagy.

[38] Hui K.Y., Fernandez-Hernandez H., Hu J., Schaffner A., Pankratz N., Hsu N.Y., Chuang L.S., Carmi S., Villaverde N., Li X. (2018). Functional variants in the LRRK2 gene confer shared effects on risk for Crohn’s disease and Parkinson’s disease. Sci. Transl. Med..

[39] Lahiri A., Hedl M., Abraham C. (2015). MTMR3 risk allele enhances innate receptor-induced signaling and cytokines by decreasing autophagy and increasing caspase-1 activation. Proc. Natl. Acad. Sci. USA.

[40] Ellinghaus D., Zhang H., Zeissig S., Lipinski S., Till A., Jiang T., Stade B., Bromberg Y., Ellinghaus E., Keller A. (2013). Association between variants of PRDM1 and NDP52 and Crohn’s disease, based on exome sequencing and functional studies. Gastroenterology.

[41] Franco L.H., Nair V.R., Scharn C.R., Xavier R.J., Torrealba J.R., Shiloh M.U., Levine B. (2017). The Ubiquitin Ligase Smurf1 Functions in Selective Autophagy of Mycobacterium tuberculosis and Anti-tuberculous Host Defense. Cell Host Microbe.

[42] Molineros J.E., Yang W., Zhou X.J., Sun C., Okada Y., Zhang H., Heng Chua K., Lau Y.L., Kochi Y., Suzuki A. (2017). Confirmation of five novel susceptibility loci for systemic lupus erythematosus (SLE) and integrated network analysis of 82 SLE susceptibility loci. Hum. Mol. Genet..

[43] Liu X., Qin H., Xu J. (2016). The role of autophagy in the pathogenesis of systemic lupus erythematosus. Int. Immunopharmacol..

[44] Rai S., Arasteh M., Jefferson M., Pearson T., Wang Y., Zhang W., Bicsak B., Divekar D., Powell P.P., Naumann R. (2019). The ATG5-binding and coiled coil domains of ATG16L1 maintain autophagy and tissue homeostasis in mice independently of the WD domain required for LC3-associated phagocytosis. Autophagy.

[45] Jin J., Chou C., Lima M., Zhou D., Zhou X. (2014). Systemic Sclerosis is a Complex Disease Associated Mainly with Immune Regulatory and Inflammatory Genes. Open Rheumatol. J..

[46] Bronson P.G., Chang D., Bhangale T., Seldin M.F., Ortmann W., Ferreira R.C., Urcelay E., Pereira L.F., Martin J., Plebani A. (2016). Common variants at PVT1, ATG13-AMBRA1, AHI1 and CLEC16A are associated with selective IgA deficiency. Nat. Genet..

[47] Schuster C., Gerold K.D., Schober K., Probst L., Boerner K., Kim M.J., Ruckdeschel A., Serwold T., Kissler S. (2015). The Autoimmunity-Associated Gene CLEC16A Modulates Thymic Epithelial Cell Autophagy and Alters T Cell Selection. Immunity.

[48] Soleimanpour S.A., Gupta A., Bakay M., Ferrari A.M., Groff D.N., Fadista J., Spruce L.A., Kushner J.A., Groop L., Seeholzer S.H. (2014). The diabetes susceptibility gene Clec16a regulates mitophagy. Cell.

[49] Scharl M., Wojtal K.A., Becker H.M., Fischbeck A., Frei P., Arikkat J., Pesch T., Kellermeier S., Boone D.L., Weber A. (2012). Protein tyrosine phosphatase nonreceptor type 2 regulates autophagosome formation in human intestinal cells. Inflamm. Bowel Dis..

[50] Kimura T., Jain A., Choi S.W., Mandell M.A., Schroder K., Johansen T., Deretic V. (2015). TRIM-mediated precision autophagy targets cytoplasmic regulators of innate immunity. J. Cell Biol..

[51] Li C.J., Liao W.T., Wu M.Y., Chu P.Y. (2017). New insights into the role of autophagy in tumor immune microenvironment. Int. J. Mol. Sci..

[52] Sahni S., Merlot A.M., Krishan S., Jansson P.J., Richardson D.R. (2014). Gene of the month: BECN1. J. Clin. Pathol..

[53] Laddha S.V., Ganesan S., Chan C.S., White E. (2014). Mutational landscape of the essential autophagy gene BECN1 in human cancers. Mol. Cancer Res..

[54] Gong Y., Zack T.I., Morris L.G., Lin K., Hukkelhoven E., Raheja R., Tan I.L., Turcan S., Veeriah S., Meng S. (2014). Pan-cancer genetic analysis identifies PARK2 as a master regulator of G1/S cyclins. Nat. Genet..

[55] Moscat J., Diaz-Meco M.T. (2012). p62, a versatile multitasker takes on cancer. Trends Biochem. Sci..

[56] Sun K., Xu L., Jing Y., Han Z., Chen X., Cai C., Zhao P., Zhao X., Yang L., Wei L. (2017). Autophagy-deficient Kupffer cells promote tumorigenesis by enhancing mtROS-NF-κB-IL1α/β-dependent inflammation and fibrosis during the preneoplastic stage of hepatocarcinogenesis. Cancer Lett..

[57] Yang A., Rajeshkumar N.V., Wang X., Yabuuchi S., Alexander B.M., Chu G.C., Von Hoff D.D., Maitra A., Kimmelman A.C. (2014). Autophagy is critical for pancreatic tumor growth and progression in tumors with p53 alterations. Cancer Discov..

[58] Dimberg L.Y., Anderson C.K., Camidge R., Behbakht K., Thorburn A., Ford H.L. (2013). On the TRAIL to successful cancer therapy? Predicting and counteracting resistance against TRAIL-based therapeutics. Oncogene.

[59] Thorburn A., Thamm D.H., Gustafson D.L. (2014). Autophagy and cancer therapy. Mol. Pharmacol..

[60] Lee D.H., Nam Y.J., Kim Y.J., Lee M.W., Lee C.S. (2014). Rotundarpene prevents TRAIL-induced apoptosis in human keratinocytes by suppressing the caspase-8- and Bid-pathways and the mitochondrial pathway. Naunyn. Schmiedebergs Arch. Pharmacol..

[61] Catalano M., D’Alessandro G., Lepore F., Corazzari M., Caldarola S., Valacca C., Faienza F., Esposito V., Limatola C., Cecconi F. (2015). Autophagy induction impairs migration and invasion by reversing EMT in glioblastoma cells. Mol. Oncol..

[62] Lv Q., Hua F., Hu Z.-W. (2012). DEDD, a novel tumor repressor, reverses epithelial-mesenchymal transition by activating selective autophagy. Autophagy.

[63] White E., Mehnert J.M., Chan C.S. (2015). Autophagy, Metabolism, and Cancer. Clin. Cancer Res..

[64] Guo J.Y., Xia B., White E. (2013). Autophagy-mediated tumor promotion. Cell.

[65] Rosenfeldt M.T., O’Prey J., Morton J.P., Nixon C., MacKay G., Mrowinska A., Au A., Rai T.S., Zheng L., Ridgway R. (2013). p53 status determines the role of autophagy in pancreatic tumour development. Nature.

[66] Rao S., Tortola L., Perlot T., Wirnsberger G., Novatchkova M., Nitsch R., Sykacek P., Frank L., Schramek D., Komnenovic V. (2014). A dual role for autophagy in a murine model of lung cancer. Nat. Commun..

[67] Stephenson Clarke J.R., Douglas L.R., Duriez P.J., Balourdas D.I., Joerger A.C., Khadiullina R., Bulatov E., Baud M.G.J. (2022). Discovery of Nanomolar-Affinity Pharmacological Chaperones Stabilizing the Oncogenic p53 Mutant Y220C. ACS Pharmacol. Transl. Sci..

[68] Wang X., Lee J., Xie C. (2022). Autophagy Regulation on Cancer Stem Cell Maintenance, Metastasis, and Therapy Resistance. Cancers.

[69] Han Y., Fan S., Qin T., Yang J., Sun Y., Lu Y., Mao J., Li L. (2018). Role of autophagy in breast cancer and breast cancer stem cells (Review). Int. J. Oncol..

[70] Song Y.J., Zhang S.S., Guo X.L., Sun K., Han Z.P., Li R., Zhao Q.D., Deng W.J., Xie X.Q., Zhang J.W. (2013). Autophagy contributes to the survival of CD133+ liver cancer stem cells in the hypoxic and nutrient-deprived tumor microenvironment. Cancer Lett..

[71] Peng Q., Qin J., Zhang Y., Cheng X., Wang X., Lu W., Xie X., Zhang S. (2017). Autophagy maintains the stemness of ovarian cancer stem cells by FOXA2. J. Exp. Clin. Cancer Res..

[72] Yeo S.K., Wen J., Chen S., Guan J.L. (2016). Autophagy Differentially Regulates Distinct Breast Cancer Stem-like Cells in Murine Models via EGFR/Stat3 and Tgfβ/Smad Signaling. Cancer Res..

[73] Maycotte P., Jones K.L., Goodall M.L., Thorburn J., Thorburn A. (2015). Autophagy Supports Breast Cancer Stem Cell Maintenance by Regulating IL6 Secretion. Mol. Cancer Res..

[74] Zhu H., Wang D., Liu Y., Su Z., Zhang L., Chen F., Zhou Y., Wu Y., Yu M., Zhang Z. (2013). Role of the Hypoxia-inducible factor-1 alpha induced autophagy in the conversion of non-stem pancreatic cancer cells into CD133+ pancreatic cancer stem-like cells. Cancer Cell Int..

[75] García R.O., Sánchez-Maldonado J.M., López-Nevot M.Á., García P., Macauda A., Hernández-Mohedo F., González-Sierra P.A., Martínez-Bueno M., Pérez E., Reyes-Zurita F.J. (2022). Autophagy in Hematological Malignancies. Cancers.

[76] Nascimento C., Ferreira F. (2021). Tumor microenvironment of human breast cancer, and feline mammary carcinoma as a potential study model. Biochim. Biophys. Acta Rev. Cancer.

[77] Marcucci F., Ghezzi P., Rumio C. (2017). The role of autophagy in the cross-talk between epithelial-mesenchymal transitioned tumor cells and cancer stem-like cells. Mol. Cancer.

[78] Galavotti S., Bartesaghi S., Faccenda D., Shaked-Rabi M., Sanzone S., McEvoy A., Dinsdale D., Condorelli F., Brandner S., Campanella M. (2013). The autophagy-associated factors DRAM1 and p62 regulate cell migration and invasion in glioblastoma stem cells. Oncogene.

[79] Sui X., Chen R., Wang Z., Huang Z., Kong N., Zhang M., Han W., Lou F., Yang J., Zhang Q. (2013). Autophagy and chemotherapy resistance: A promising therapeutic target for cancer treatment. Cell Death Dis..

[80] Martelli A., Omrani M., Zarghooni M., Citi V., Brogi S., Calderone V., Sureda A., Lorzadeh S., da Silva Rosa S.C., Grabarek B.O. (2022). New Visions on Natural Products and Cancer Therapy: Autophagy and Related Regulatory Pathways. Cancers.

[81] Sun R., Shen S., Zhang Y.J., Xu C.F., Cao Z.T., Wen L.P., Wang J. (2016). Nanoparticle-facilitated autophagy inhibition promotes the efficacy of chemotherapeutics against breast cancer stem cells. Biomaterials.

[82] Viry E., Paggetti J., Baginska J., Mgrditchian T., Berchem G., Moussay E., Janji B. (2014). Autophagy: An adaptive metabolic response to stress shaping the antitumor immunity. Biochem. Pharmacol..

[83] El Hout M., Cosialls E., Mehrpour M., Hamaï A. (2020). Crosstalk between autophagy and metabolic regulation of cancer stem cells. Mol. Cancer.

[84] Boya P., Codogno P., Rodriguez-Muela N. (2018). Autophagy in stem cells: Repair, remodelling and metabolic reprogramming. Development.

[85] Yan C., Luo L., Guo C.Y., Goto S., Urata Y., Shao J.H., Li T.S. (2017). Doxorubicin-induced mitophagy contributes to drug resistance in cancer stem cells from HCT8 human colorectal cancer cells. Cancer Lett..

[86] Duan Z., Shi Y., Lin Q., Hamaï A., Mehrpour M., Gong C. (2022). Autophagy-Associated Immunogenic Modulation and Its Applications in Cancer Therapy. Cells.

[87] Jacquel A., Obba S., Boyer L., Dufies M., Robert G., Gounon P., Lemichez E., Luciano F., Solary E., Auberger P. (2012). Autophagy is required for CSF-1-induced macrophagic differentiation and acquisition of phagocytic functions. Blood.

[88] Roca H., Varsos Z.S., Sud S., Craig M.J., Ying C., Pienta K.J. (2009). CCL2 and interleukin-6 promote survival of human CD11b+ peripheral blood mononuclear cells and induce M2-type macrophage polarization. J. Biol. Chem..

[89] Jin Z., Sun X., Wang Y., Zhou C., Yang H., Zhou S. (2022). Regulation of autophagy fires up the cold tumor microenvironment to improve cancer immunotherapy. Front. Immunol..

[90] Biswas S.K., Lewis C.E. (2010). NF-κB as a central regulator of macrophage function in tumors. J. Leukoc. Biol..

[91] Ngabire D., Kim G.D. (2017). Autophagy and Inflammatory Response in the Tumor Microenvironment. Int. J. Mol. Sci..

[92] Martinez-Outschoorn U.E., Whitaker-Menezes D., Lin Z., Flomenberg N., Howell A., Pestell R.G., Lisanti M.P., Sotgia F. (2011). Cytokine production and inflammation drive autophagy in the tumor microenvironment: Role of stromal caveolin-1 as a key regulator. Cell Cycle.

[93] Martinez-Outschoorn U.E., Trimmer C., Lin Z., Whitaker-Menezes D., Chiavarina B., Zhou J., Wang C., Pavlides S., Martinez-Cantarin M.P., Capozza F. (2010). Autophagy in cancer associated fibroblasts promotes tumor cell survival: Role of hypoxia, HIF1 induction and NFκB activation in the tumor stromal microenvironment. Cell Cycle.

[94] Capparelli C., Guido C., Whitaker-Menezes D., Bonuccelli G., Balliet R., Pestell T.G., Goldberg A.F., Pestell R.G., Howell A., Sneddon S. (2012). Autophagy and senescence in cancer-associated fibroblasts metabolically supports tumor growth and metastasis via glycolysis and ketone production. Cell Cycle.

[95] Műzes G., Bohusné Barta B., Szabó O., Horgas V., Sipos F. (2022). Cell-Free DNA in the Pathogenesis and Therapy of Non-Infectious Inflammations and Tumors. Biomedicines.

[96] Sipos F., Kiss A.L., Constantinovits M., Tulassay Z., Műzes G. (2019). Modified Genomic Self-DNA Influences In Vitro Survival of HT29 Tumor Cells via TLR9- and Autophagy Signaling. Pathol. Oncol. Res..

[97] Sipos F., Bohusné Barta B., Simon Á., Nagy L., Dankó T., Raffay R.E., Petővári G., Zsiros V., Wichmann B., Sebestyén A. (2022). Survival of HT29 Cancer Cells Is Affected by IGF1R Inhibition via Modulation of Self-DNA-Triggered TLR9 Signaling and the Autophagy Response. Pathol. Oncol. Res..

[98] Qi Y., Zou H., Zhao X., Kapeleris J., Monteiro M., Li F., Xu Z.P., Deng Y., Wu Y., Tang Y. (2022). Inhibition of colon cancer K-RasG13D mutation reduces cancer cell proliferation but promotes stemness and inflammation via RAS/ERK pathway. Front. Pharmacol..

[99] Kaźmierczak-Siedlecka K., Daca A., Fic M., van de Wetering T., Folwarski M., Makarewicz W. (2020). Therapeutic methods of gut microbiota modification in colorectal cancer management—fecal microbiota transplantation, prebiotics, probiotics, and synbiotics. Gut Microbes.

[100] Kostic A.D., Chun E., Robertson L., Glickman J.N., Gallini C.A., Michaud M., Clancy T.E., Chung D.C., Lochhead P., Hold G.L. (2013). Fusobacterium nucleatum potentiates intestinal tumorigenesis and modulates the tumor-immune microenvironment. Cell Host Microbe.

[101] Mäkinen A., Nawaz A., Mäkitie A., Meurman J.H. (2018). Role of Non-Albicans Candida and Candida Albicans in Oral Squamous Cell Cancer Patients. J. Oral Maxillofac. Surg..

[102] Kaźmierczak-Siedlecka K., Daca A., Roviello G., Catalano M., Połom K. (2022). Interdisciplinary insights into the link between gut microbiome and gastric carcinogenesis-what is currently known?. Gastric Cancer.

[103] Bakhti S.Z., Latifi-Navid S. (2021). Interplay and cooperation of Helicobacter pylori and gut microbiota in gastric carcinogenesis. BMC Microbiol..

[104] Matson V., Chervin C.S., Gajewski T.F. (2021). Cancer and the Microbiome—Influence of the Commensal Microbiota on Cancer, Immune Responses, and Immunotherapy. Gastroenterology.

[105] Li W., Deng Y., Chu Q., Zhang P. (2019). Gut microbiome and cancer immunotherapy. Cancer Lett..

[106] Routy B., Le Chatelier E., Derosa L., Duong C.P.M., Alou M.T., Daillère R., Fluckiger A., Messaoudene M., Rauber C., Roberti M.P. (2018). Gut microbiome influences efficacy of PD-1-based immunotherapy against epithelial tumors. Science.

[107] Michail S., Bultron G., Depaolo R.W. (2013). Genetic variants associated with Crohn’s disease. Appl. Clin. Genet..

[108] Caparrós E., Wiest R., Scharl M., Rogler G., Gutiérrez Casbas A., Yilmaz B., Wawrzyniak M., Francés R. (2021). Dysbiotic microbiota interactions in Crohn’s disease. Gut Microbes.

[109] Ott S.J., Musfeldt M., Wenderoth D.F., Hampe J., Brant O., Fölsch U.R., Timmis K.N., Schreiber S. (2004). Reduction in diversity of the colonic mucosa associated bacterial microflora in patients with active inflammatory bowel disease. Gut.

[110] Wagner J., Skinner N.A., Catto-Smith A.G., Cameron D.J.S., Michalski W.P., Visvanathan K., Kirkwood C.D. (2013). TLR4, IL10RA, and NOD2 mutation in paediatric Crohn’s disease patients: An association with Mycobacterium avium subspecies paratuberculosis and TLR4 and IL10RA expression. Med. Microbiol. Immunol..

[111] Hugot J.P., Chamaillard M., Zouali H., Lesage S., Cézard J.P., Belaiche J., Almer S., Tysk C., O’Morain C.A., Gassull M. (2001). Association of NOD2 leucine-rich repeat variants with susceptibility to Crohn’s disease. Nature.

[112] Abdelnaby H., Ndiaye N., D’Amico F., Fouad A., Hassan S., Elshafey A., Al Hashash W., Faisal M., Alshamali Y., Al-Taweel T. (2021). NOD2/CARD15 polymorphisms (P268S, IVS8+158, G908R, L1007fs, R702W) among Kuwaiti patients with Crohn’s disease: A case-control study. Saudi J. Gastroenterol..

[113] Smatti M.K., Cyprian F.S., Nasrallah G.K., Al Thani A.A., Almishal R.O., Yassine H.M. (2019). Viruses and Autoimmunity: A Review on the Potential Interaction and Molecular Mechanisms. Viruses.

[114] Coppieters K.T., Wiberg A., von Herrath M.G. (2012). Viral infections and molecular mimicry in type 1 diabetes. APMIS.

[115] Rojas M., Restrepo-Jiménez P., Monsalve D.M., Pacheco Y., Acosta-Ampudia Y., Ramírez-Santana C., Leung P.S.C., Ansari A.A., Gershwin M.E., Anaya J.M. (2018). Molecular mimicry and autoimmunity. J. Autoimmun..

[116] Rendell M.S. (2023). Obesity and diabetes: The final frontier. Expert Rev. Endocrinol. Metab..

[117] An C., Pipia I., Ruiz A.S., Argüelles I., An M., Wase S., Peng G. (2023). The molecular link between obesity and genomic instability in cancer development. Cancer Lett..

[118] Cao Y., Ren G., Zhang Y., Qin H., An X., Long Y., Chen J., Yang L. (2021). A new way for punicalagin to alleviate insulin resistance: Regulating gut microbiota and autophagy. Food Nutr. Res..

[119] Ceccarelli F., Agmon-Levin N., Perricone C. (2017). Genetic Factors of Autoimmune Diseases 2017. J. Immunol. Res..

[120] Noble J.A., Valdes A.M. (2011). Genetics of the HLA region in the prediction of type 1 diabetes. Curr. Diab Rep..

[121] Kulski J.K., Suzuki S., Shiina T. (2022). Human leukocyte antigen super-locus: Nexus of genomic supergenes, SNPs, indels, transcripts, and haplotypes. Hum. Genome Var..

[122] Das P., Abraham R., David C. (2000). HLA transgenic mice as models of human autoimmune diseases. Rev. Immunogenetics.

[123] Rouanne M., Adam J., Radulescu C., Letourneur D., Bredel D., Mouraud S., Goubet A.G., Leduc M., Chen N., Tan T.Z. (2022). BCG therapy downregulates HLA-I on malignant cells to subvert antitumor immune responses in bladder cancer. J. Clin. Invest..

[124] Mehra P., Wells A.D. (2021). Variant to Gene Mapping to Discover New Targets for Immune Tolerance. Front. Immunol..

[125] Musette P., Bouaziz J.D. (2018). B Cell Modulation Strategies in Autoimmune Diseases: New Concepts. Front. Immunol..

[126] Anderson M.S., Venanzi E.S., Chen Z., Berzins S.P., Benoist C., Mathis D. (2005). The cellular mechanism of Aire control of T cell tolerance. Immunity.

[127] Fierabracci A. (2011). Recent insights into the role and molecular mechanisms of the autoimmune regulator (AIRE) gene in autoimmunity. Autoimmun. Rev..

[128] Perniola R. (2018). Twenty Years of AIRE. Front. Immunol..

[129] Zhu M.L., Bakhru P., Conley B., Nelson J.S., Free M., Martin A., Starmer J., Wilson E.M., Su M.A. (2016). Sex bias in CNS autoimmune disease mediated by androgen control of autoimmune regulator. Nat. Commun..

[130] Dragin N., Bismuth J., Cizeron-Clairac G., Biferi M.G., Berthault C., Serraf A., Nottin R., Klatzmann D., Cumano A., Barkats M. (2016). Estrogen-mediated downregulation of AIRE influences sexual dimorphism in autoimmune diseases. J. Clin. Invest..

[131] Shi L., Hu L.H., Li Y.R. (2010). Autoimmune regulator regulates autophagy in THP-1 human monocytes. Front. Med. China.

[132] Rodrigues P.M., Sousa L.G., Perrod C., Maceiras A.R., Ferreirinha P., Pombinho R., Romera-Cárdenas G., Gomez-Lazaro M., Senkara M., Pistolic J. (2023). LAMP2 regulates autophagy in the thymic epithelium and thymic stroma-dependent CD4 T cell development. Autophagy.

[133] Alessandrini F., Pezzè L., Ciribilli Y. (2017). LAMPs: Shedding light on cancer biology. Semin. Oncol..

[134] Liu S.-P., Li X.-M., Liu D.-M., Xie S.-H., Zhang S.-B., Li Y., Xie Z.-F. (2022). LAMP2 as a Biomarker Related to Prognosis and Immune Infiltration in Esophageal Cancer and Other Cancers: A Comprehensive Pan-Cancer Analysis. Front. Oncol..

[135] Kravtsov D.S., Erbe A.K., Sondel P.M., Rakhmilevich A.L. (2022). Roles of CD4+ T cells as mediators of antitumor immunity. Front. Immunol..

[136] Greisen S.R., Aspari M., Deleuran B. (2022). Co-inhibitory molecules – their role in health and autoimmunity; highlighted by immune related adverse events. Front. Immunol..

[137] Cubas R., Khan Z., Gong Q., Moskalenko M., Xiong H., Ou Q., Pai C., Rodriguez R., Cheung J., Chan A.C. (2020). Autoimmunity linked protein phosphatase PTPN22 as a target for cancer immunotherapy. J. Immunother. Cancer.

[138] Arechiga A.F., Habib T., He Y., Zhang X., Zhang Z.Y., Funk A., Buckner J.H. (2009). Cutting edge: The PTPN22 allelic variant associated with autoimmunity impairs B cell signaling. J. Immunol..

[139] Begovich A.B., Carlton V.E., Honigberg L.A., Schrodi S.J., Chokkalingam A.P., Alexander H.C., Ardlie K.G., Huang Q., Smith A.M., Spoerke J.M. (2004). A missense single-nucleotide polymorphism in a gene encoding a protein tyrosine phosphatase (PTPN22) is associated with rheumatoid arthritis. Am. J. Hum. Genet..

[140] Velaga M.R., Wilson V., Jennings C.E., Owen C.J., Herington S., Donaldson P.T., Ball S.G., James R.A., Quinton R., Perros P. (2004). The codon 620 tryptophan allele of the lymphoid tyrosine phosphatase (LYP) gene is a major determinant of Graves’ disease. J. Clin. Endocrinol. Metab..

[141] Smyth D., Cooper J.D., Collins J.E., Heward J.M., Franklyn J.A., Howson J.M., Vella A., Nutland S., Rance H.E., Maier L. (2004). Replication of an association between the lymphoid tyrosine phosphatase locus (LYP/PTPN22) with type 1 diabetes, and evidence for its role as a general autoimmunity locus. Diabetes.

[142] Bottini N., Musumeci L., Alonso A., Rahmouni S., Nika K., Rostamkhani M., MacMurray J., Meloni G.F., Lucarelli P., Pellecchia M. (2004). A functional variant of lymphoid tyrosine phosphatase is associated with type I diabetes. Nat. Genet..

[143] Ho W.J., Croessmann S., Lin J., Phyo Z.H., Charmsaz S., Danilova L., Mohan A.A., Gross N.E., Chen F., Dong J. (2021). Systemic inhibition of PTPN22 augments anticancer immunity. J. Clin. Invest..

[144] Martyna B., Małgorzata M.W., Nikola Z., Beniamin G., Urszula M., Grażyna J. (2019). Expression Profile of Genes Associated with the Proteins Degradation Pathways in Colorectal adenocarcinoma. Curr. Pharm. Biotechnol..

[145] Kovats S. (2015). Estrogen receptors regulate innate immune cells and signaling pathways. Cell Immunol..

[146] Maglione A., Rolla S., Mercanti S.F., Cutrupi S., Clerico M. (2019). The Adaptive Immune System in Multiple Sclerosis: An Estrogen-Mediated Point of View. Cells.

[147] Tai P., Wang J., Jin H., Song X., Yan J., Kang Y., Zhao L., An X., Du X., Chen X. (2008). Induction of regulatory T cells by physiological level estrogen. J. Cell Physiol..

[148] Hill L., Jeganathan V., Chinnasamy P., Grimaldi C., Diamond B. (2011). Differential roles of estrogen receptors α and β in control of B-cell maturation and selection. Mol. Med..

[149] Williams M.M., Spoelstra N.S., Arnesen S., O’Neill K.I., Christenson J.L., Reese J., Torkko K.C., Goodspeed A., Rosas E., Hanamura T. (2021). Steroid Hormone Receptor and Infiltrating Immune Cell Status Reveals Therapeutic Vulnerabilities of ESR1-Mutant Breast Cancer. Cancer Res..

[150] Dou C., Ding N., Zhao C., Hou T., Kang F., Cao Z., Liu C., Bai Y., Dai Q., Ma Q. (2018). Estrogen Deficiency-Mediated M2 Macrophage Osteoclastogenesis Contributes to M1/M2 Ratio Alteration in Ovariectomized Osteoporotic Mice. J. Bone. Miner. Res..

[151] Campbell L., Emmerson E., Williams H., Saville C.R., Krust A., Chambon P., Mace K.A., Hardman M.J. (2014). Estrogen receptor-alpha promotes alternative macrophage activation during cutaneous repair. J. Investig. Dermatol..

[152] Ranhotra H.S. (2022). Estrogen-related receptor alpha in select host functions and cancer: New frontiers. Mol. Cell Biochem..

[153] Xiang J., Liu X., Ren J., Chen K., Wang H.L., Miao Y.Y., Qi M.M. (2019). How does estrogen work on autophagy?. Autophagy.

[154] De Sousa Linhares A., Leitner J., Grabmeier-Pfistershammer K., Steinberger P. (2018). Not All Immune Checkpoints Are Created Equal. Front. Immunol..

[155] Tivol E.A., Borriello F., Schweitzer A.N., Lynch W.P., Bluestone J.A., Sharpe A.H. (1995). Loss of CTLA-4 leads to massive lymphoproliferation and fatal multiorgan tissue destruction, revealing a critical negative regulatory role of CTLA-4. Immunity.

[156] Wong C.K., Lam T.H., Liao S.Y., Lau Y.M., Tse H.F., So B.Y.F. (2023). Immunopathogenesis of Immune Checkpoint Inhibitor Induced Myocarditis: Insights from Experimental Models and Treatment Implications. Biomedicines.

[157] Nishimura H., Nose M., Hiai H., Minato N., Honjo T. (1999). Development of lupus-like autoimmune diseases by disruption of the PD-1 gene encoding an ITIM motif-carrying immunoreceptor. Immunity.

[158] Oya Y., Watanabe N., Kobayashi Y., Owada T., Oki M., Ikeda K., Suto A., Kagami S., Hirose K., Kishimoto T. (2011). Lack of B and T lymphocyte attenuator exacerbates autoimmune disorders and induces Fas-independent liver injury in MRL-lpr/lpr mice. Int. Immunol..

[159] Wang L., Le Mercier I., Putra J., Chen W., Liu J., Schenk A.D., Nowak E.C., Suriawinata A.A., Li J., Noelle R.J. (2014). Disruption of the immune-checkpoint VISTA gene imparts a proinflammatory phenotype with predisposition to the development of autoimmunity. Proc. Natl. Acad. Sci. USA.

[160] Joller N., Hafler J.P., Brynedal B., Kassam N., Spoerl S., Levin S.D., Sharpe A.H., Kuchroo V.K. (2011). Cutting edge: TIGIT has T cell-intrinsic inhibitory functions. J. Immunol..

[161] Dörner T., Szelinski F., Lino A.C., Lipsky P.E. (2020). Therapeutic implications of the anergic/postactivated status of B cells in systemic lupus erythematosus. RMD Open.

[162] Yu L., Shao M., Zhou T., Xie H., Wang F., Kong J., Xu S., Shuai Z., Pan F. (2021). Association of CTLA-4 (+49 A/G) polymorphism with susceptibility to autoimmune diseases: A meta-analysis with trial sequential analysis. Int. Immunopharmacol..

[163] Chen S., Li Y., Deng C., Li J., Wen X., Wu Z., Hu C., Zhang S., Li P., Zhang X. (2016). The associations between PD-1, CTLA-4 gene polymorphisms and susceptibility to ankylosing spondylitis: A meta-analysis and systemic review. Rheumatol Int..

[164] Liu R., Wang X., Chen X., Wang S., Zhang H. (2018). TIM-3 rs1036199 polymorphism increases susceptibility to autoimmune diseases: Evidence based on 4200 subjects. Biosci. Rep..

[165] Pawlak-Adamska E., Nowak O., Karabon L., Pokryszko-Dragan A., Partyka A., Tomkiewicz A., Ptaszkowski J., Frydecka I., Podemski R., Dybko J. (2017). Bilinska MPD-1 gene polymorphic variation is linked with first symptom of disease severity of relapsing-remitting form of MS. J. Neuroimmunol..

[166] Rudd C.E., Taylor A., Schneider H. (2009). CD28 and CTLA-4 coreceptor expression and signal transduction. Immunol. Rev..

[167] Qureshi O.S., Zheng Y., Nakamura K., Attridge K., Manzotti C., Schmidt E.M., Baker J., Jeffery L.E., Kaur S., Briggs Z. (2011). Trans-endocytosis of CD80 and CD86, a molecular basis for the cell-extrinsic function of CTLA-4. Science.

[168] Jain N., Nguyen H., Chambers C., Kang J. (2010). Dual function of CTLA-4 in regulatory T cells and conventional T cells to prevent multiorgan autoimmunity. Proc. Natl. Acad. Sci. USA.

[169] Verma N., Burns S.O., Walker L.S.K., Sansom D.M. (2017). Immune deficiency and autoimmunity in patients with CTLA-4 (CD152) mutations. Clin. Exp. Immunol..

[170] Zhang B., Dang J., Ba D., Wang C., Han J., Zheng F. (2018). Potential function of CTLA-4 in the tumourigenic capacity of melanoma stem cells. Oncol. Lett..

[171] Roncella S., Laurent S., Fontana V., Ferro P., Franceschini M.C., Salvi S., Varesano S., Boccardo S., Vigani A., Morabito A. (2016). CTLA-4 in mesothelioma patients: Tissue expression, body fluid levels and possible relevance as a prognostic factor. Cancer Immunol. Immunother..

[172] Zhao Y., Yang W., Huang Y., Cui R., Li X., Li B. (2018). Evolving Roles for Targeting CTLA-4 in Cancer Immunotherapy. Cell Physiol. Biochem..

[173] Urbano A.C., Nascimento C., Soares M., Correia J., Ferreira F. (2020). Clinical Relevance of the serum CTLA-4 in Cats with Mammary Carcinoma. Sci. Rep..

[174] Romano E., Kusio-Kobialka M., Foukas P.G., Baumgaertner P., Meyer C., Ballabeni P., Michielin O., Weide B., Romero P., Speiser D.E. (2015). Ipilimumab-dependent cell-mediated cytotoxicity of regulatory T cells ex vivo by nonclassical monocytes in melanoma patients. Proc. Natl. Acad. Sci. USA.

[175] Sharma A., Subudhi S.K., Blando J., Scutti J., Vence L., Wargo J., Allison J.P., Ribas A., Sharma P. (2019). Anti-CTLA-4 Immunotherapy Does Not Deplete Foxp3 Þ Regulatory T Cells (Tregs) in Human Cancers. Clin. Cancer Res..

[176] Shukla S.A., Bachireddy P., Schilling B., Galonska C., Zhan Q., Bango C., Langer R., Lee P.C., Gusenleitner D., Keskin D.B. (2018). Cancer-Germline Antigen Expression Discriminates Clinical Outcome to CTLA-4 Blockade. Cell.

[177] Jiang G.M., Tan Y., Wang H., Peng L., Chen H.T., Meng X.J., Li L.L., Liu Y., Li W.F., Shan H. (2019). The relationship between autophagy and the immune system and its applications for tumor immunotherapy. Mol. Cancer.

[178] Pauken K.E., Jenkins M.K., Azuma M., Fife B.T. (2013). PD-1, but not PD-L1, expressed by islet-reactive CD4+ T cells suppresses infiltration of the pancreas during type 1 diabetes. Diabetes.

[179] Ke Y., Sun D., Jiang G., Kaplan H.J., Shao H. (2010). PD-L1(hi) retinal pigment epithelium (RPE) cells elicited by inflammatory cytokines induce regulatory activity in uveitogenic T cells. J. Leukoc. Biol..

[180] Keir M.E., Liang S.C., Guleria I., Latchman Y.E., Qipo A., Albacker L.A., Koulmanda M., Freeman G.J., Sayegh M.H., Sharpe A.H. (2006). Tissue expression of PD-L1 mediates peripheral T cell tolerance. J. Exp. Med..

[181] Colli M.L., Hill J.L.E., Marroquí L., Chaffey J., Dos Santos R.S., Leete P., Coomans de Brachène A., Paula F.M.M., Op de Beeck A., Castela A. (2018). PDL1 is expressed in the islets of people with type 1 diabetes and is up-regulated by interferons-α and-γ via IRF1 induction. EBioMedicine.

[182] Osum K.C., Burrack A.L., Martinov T., Sahli N.L., Mitchell J.S., Tucker C.G., Pauken K.E., Papas K., Appakalai B., Spanier J.A. (2018). Interferon-gamma drives programmed death-ligand 1 expression on islet β cells to limit T cell function during autoimmune diabetes. Sci. Rep..

[183] Abdeladhim M., Karnell J.L., Rieder S.A. (2022). In or out of control: Modulating regulatory T cell homeostasis and function with immune checkpoint pathways. Front. Immunol..

[184] Zhao Z., Wang X., Bao X.Q., Ning J., Shang M., Zhang D. (2021). Autoimmune polyendocrine syndrome induced by immune checkpoint inhibitors: A systematic review. Cancer Immunol. Immunother..

[185] Schneider S., Potthast S., Komminoth P., Schwegler G., Böhm S. (2017). PD-1 Checkpoint Inhibitor Associated Autoimmune Encephalitis. Case Rep. Oncol..

[186] Hakroush S., Tampe B. (2023). Association between Loss of Immune Checkpoint Programmed Cell Death Protein 1 and Active ANCA-Associated Renal Vasculitis. Int. J. Mol. Sci..

[187] Parry R.V., Chemnitz J.M., Frauwirth K.A., Lanfranco A.R., Braunstein I., Kobayashi S.V., Linsley P.S., Thompson C.B., Riley J.L. (2005). CTLA-4 and PD-1 receptors inhibit T-cell activation by distinct mechanisms. Mol. Cell Biol..

[188] Patsoukis N., Bardhan K., Chatterjee P., Sari D., Liu B., Bell L.N., Karoly E.D., Freeman G.J., Petkova V., Seth P. (2015). PD-1 alters T-cell metabolic reprogramming by inhibiting glycolysis and promoting lipolysis and fatty acid oxidation. Nat. Commun..

[189] Jiang X., Wang J., Deng X., Xiong F., Ge J., Xiang B., Wu X., Ma J., Zhou M., Li X. (2019). Role of the tumor microenvironment in PD-L1/PD-1-mediated tumor immune escape. Mol. Cancer..

[190] Chen G., Huang A.C., Zhang W., Zhang G., Wu M., Xu W., Yu Z., Yang J., Wang B., Sun H. (2018). Exosomal PD-L1 contributes to immunosuppression and is associated with anti-PD-1 response. Nature.

[191] Jancewicz I., Szarkowska J., Konopinski R., Stachowiak M., Swiatek M., Blachnio K., Kubala S., Oksinska P., Cwiek P., Rusetska N. (2021). PD-L1 Overexpression, SWI/SNF Complex Deregulation, and Profound Transcriptomic Changes Characterize Cancer-Dependent Exhaustion of Persistently Activated CD4+ T Cells. Cancers.

[192] He Q.F., Xu Y., Li J., Huang Z.M., Li X.H., Wang X. (2019). CD8+ T-cell exhaustion in cancer: Mechanisms and new area for cancer immunotherapy. Brief. Funct. Genom..

[193] Robainas M., Otano R., Bueno S., Ait-Oudhia S. (2017). Understanding the role of PD-L1/PD1 pathway blockade and autophagy in cancer therapy. OncoTargets Ther..

[194] Maher C.M., Thomas J.D., Haas D.A., Longen C.G., Oyer H.M., Tong J.Y., Kim F.J. (2018). Small-Molecule Sigma1 Modulator Induces Autophagic Degradation of PD-L1. Mol. Cancer Res..

[195] Ashrafizadeh M., Zarrabi A., Hushmandi K., Zarrin V., Moghadam E.R., Zabolian A., Tavakol S., Samarghandian S., Najafi M. (2020). PD-1/PD-L1 axis regulation in cancer therapy: The role of long non-coding RNAs and microRNAs. Life Sci..

[196] Clark C.A., Gupta H.B., Curiel T.J. (2017). Tumor cell-intrinsic CD274/PD-L1, A novel metabolic balancing act with clinical potential. Autophagy.

[197] Cai J., Qi Q., Qian X., Han J., Zhu X., Zhang Q., Xia R. (2019). The role of PD-1/PD-L1 axis and macrophage in the progression and treatment of cancer. J. Cancer Res. Clin. Oncol..

[198] Yamada A., Arakaki R., Saito M., Kudo Y., Ishimaru N. (2017). Dual Role of Fas/FasL-Mediated Signal in Peripheral Immune Tolerance. Front. Immunol..

[199] Niederkorn J.Y. (2006). See no evil, hear no evil, do no evil: The lessons of immune privilege. Nat. Immunol..

[200] Li H., Tsokos G.C. (2021). Double-negative T cells in autoimmune diseases. Curr. Opin. Rheumatol..

[201] Rensing-Ehl A., Völkl S., Speckmann C., Lorenz M.R., Ritter J., Janda A., Abinun M., Pircher H., Bengsch B., Thimme R. (2014). Abnormally differentiated CD4+ or CD8+ T cells with phenotypic and genetic features of double negative T cells in human Fas deficiency. Blood.

[202] Saxena A., Yagita H., Donner T.W., Hamad A.R.A. (2017). Expansion of FasL-Expressing CD5+ B Cells in Type 1 Diabetes Patients. Front. Immunol..

[203] Li L., Liu S., Yu J. (2020). Autoimmune thyroid disease and type 1 diabetes mellitus: Same pathogenesis; new perspective?. Ther. Adv. Endocrinol. Metab..

[204] de Oliveira G.L., Ferreira A.F., Gasparotto E.P., Kashima S., Covas D.T., Guerreiro C.T., Brum D.G., Barreira A.A., Voltarelli J.C., Simões B.P. (2017). Defective expression of apoptosis-related molecules in multiple sclerosis patients is normalized early after autologous haematopoietic stem cell transplantation. Clin. Exp. Immunol..

[205] Hohlbaum A.M., Moe S., Marshak-Rothstein A. (2000). Opposing effects of transmembrane and soluble Fas ligand expression on inflammation and tumor cell survival. J. Exp. Med..

[206] Vincent F.B., Kandane-Rathnayake R., Koelmeyer R., Harris J., Hoi A.Y., Mackay F., Morand E.F. (2020). Associations of serum soluble Fas and Fas ligand (FasL) with outcomes in systemic lupus erythematosus. Lupus Sci. Med..

[207] Vincent F.B., Bubicich M., Downie-Doyle S., Mackay F., Morand E.F., Rischmueller M. (2019). Serum soluble Fas and Fas ligand (FasL) in primary Sjögren’s syndrome. Clin. Exp. Rheumatol..

[208] Horton B.L., Williams J.B., Cabanov A., Spranger S., Gajewski T.F. (2018). Intratumoral CD8+ T-cell Apoptosis Is a Major Component of T-cell Dysfunction and Impedes Antitumor Immunity. Cancer Immunol. Res..

[209] Zhu J., Powis de Tenbossche C.G., Cané S., Colau D., van Baren N., Lurquin C., Schmitt-Verhulst A.M., Liljeström P., Uyttenhove C., Van den Eynde B.J. (2017). Resistance to cancer immunotherapy mediated by apoptosis of tumor-infiltrating lymphocytes. Nat. Commun..

[210] Wada A., Tada Y., Kawamura K., Takiguchi Y., Tatsumi K., Kuriyama T., Takenouchi T., O-Wang J., Tagawa M. (2007). The effects of FasL on inflammation and tumor survival are dependent on its expression levels. Cancer Gene Ther..

[211] Khodapasand E., Jafarzadeh N., Farrokhi F., Kamalidehghan B., Houshmand M. (2015). Is Bax/Bcl-2 ratio considered as a prognostic marker with age and tumor location in colorectal cancer?. Iran Biomed. J..

[212] Kulsoom B., Shamsi T.S., Afsar N.A., Memon Z., Ahmed N., Hasnain S.N. (2018). Bax, Bcl-2, and Bax/Bcl-2 as prognostic markers in acute myeloid leukemia: Are we ready for Bcl-2-directed therapy?. Cancer Manag. Res..

[213] Raisova M., Hossini A.M., Eberle J., Riebeling C., Wieder T., Sturm I., Daniel P.T., Orfanos C.E., Geilen C.C. (2001). The Bax/Bcl-2 ratio determines the susceptibility of human melanoma cells to CD95/Fas-mediated apoptosis. J. Investig. Dermatol..

[214] Ploumaki I., Triantafyllou E., Koumprentziotis I.A., Karampinos K., Drougkas K., Karavolias I., Trontzas I., Kotteas E.A. (2023). Bcl-2 pathway inhibition in solid tumors: A review of clinical trials. Clin. Transl. Oncol..

[215] Mérino D., Bouillet P. (2009). The Bcl-2 family in autoimmune and degenerative disorders. Apoptosis.

[216] Strasser A., Harris A.W., Cory S. (1991). bcl-2 transgene inhibits T cell death and perturbs thymic self-censorship. Cell.

[217] Strasser A., Whittingham S., Vaux D.L., Bath M.L., Adams J.M., Cory S., Harris A.W. (1991). Enforced BCL2 expression in B-lymphoid cells prolongs antibody responses and elicits autoimmune disease. Proc. Natl. Acad. Sci. USA.

[218] Li M., Gao P., Zhang J. (2016). Crosstalk between Autophagy and Apoptosis: Potential and Emerging Therapeutic Targets for Cardiac Diseases. Int. J. Mol. Sci..

[219] Chen Y., Liu X.R., Yin Y.Q., Lee C.J., Wang F.T., Liu H.Q., Wu X.T., Liu J. (2014). Unravelling the multifaceted roles of Atg proteins to improve cancer therapy. Cell Prolif..

[220] Hounsell C., Fan Y. (2021). The Duality of Caspases in Cancer, as Told through the Fly. Int. J. Mol. Sci..

[221] You R., He X., Zeng Z., Zhan Y., Xiao Y., Xiao R. (2022). Pyroptosis and Its Role in Autoimmune Disease: A Potential Therapeutic Target. Front. Immunol..

[222] Pezone A., Olivieri F., Napoli M.V., Procopio A., Avvedimento E.V., Gabrielli A. (2023). Inflammation and DNA damage: Cause, effect or both. Nat. Rev. Rheumatol..

[223] Chen X., Zhang T., Su W., Dou Z., Zhao D., Jin X., Lei H., Wang J., Xie X., Cheng B. (2022). Mutant p53 in cancer: From molecular mechanism to therapeutic modulation. Cell Death Dis..

[224] Kataoka T. (2005). The caspase-8 modulator c-FLIP. Crit. Rev. Immunol..

[225] Seki M., Oomizu S., Sakata K.M., Sakata A., Arikawa T., Watanabe K., Ito K., Takeshita K., Niki T., Saita N. (2008). Galectin-9 suppresses the generation of Th17, promotes the induction of regulatory T cells, and regulates experimental autoimmune arthritis. Clin. Immunol..

[226] Zhu C., Anderson A.C., Schubart A., Xiong H., Imitola J., Khoury S.J., Zheng X.X., Strom T.B., Kuchroo V.K. (2005). The Tim-3 ligand galectin-9 negatively regulates T helper type 1 immunity. Nat. Immunol..

[227] Liu X., Alexiou M., Martin-Orozco N., Chung Y., Nurieva R.I., Ma L., Tian Q., Kollias G., Lu S., Graf D. (2009). Cutting edge: A critical role of B and T lymphocyte attenuator in peripheral T cell tolerance induction. J. Immunol..

[228] Wiedemann A., Lettau M., Weißenberg S.Y., Stefanski A.L., Schrezenmeier E.V., Rincon-Arevalo H., Reiter K., Alexander T., Hiepe F., Lino A.C. (2021). BTLA Expression and Function Are Impaired on SLE B Cells. Front. Immunol..

[229] Oster C., Wilde B., Specker C., Sun M., Kribben A., Witzke O., Dolff S. (2019). BTLA Expression on Th1, Th2 and Th17 Effector T-Cells of Patients with Systemic Lupus Erythematosus Is Associated with Active Disease. Int. J. Mol. Sci..

[230] Piancone F., Saresella M., Marventano I., La Rosa F., Zoppis M., Agostini S., Longhi R., Caputo D., Mendozzi L., Rovaris M. (2016). B Lymphocytes in Multiple Sclerosis: Bregs and BTLA/CD272 Expressing-CD19+ Lymphocytes Modulate Disease Severity. Sci. Rep..

[231] Liu J., Ming S., Song W., Meng X., Xiao Q., Wu M., Wu Y., Xie H., Zhou J., Zhong H. (2021). B and T lymphocyte attenuator regulates autophagy in mycobacterial infection via the AKT/mTOR signal pathway. Int. Immunopharmacol..

[232] Cheng T.Y., Liu Y.J., Yan H., Xi Y.B., Duan L.Q., Wang Y., Zhang T.T., Gu Y.M., Wang X.D., Wu C.X. (2022). Tumor Cell-Intrinsic BTLA Receptor Inhibits the Proliferation of Tumor Cells via ERK1/2. Cells.

[233] Chen M.M., Xiao X., Lao X.M., Wei Y., Liu R.X., Zeng Q.H., Wang J.C., Ouyang F.Z., Chen D.P., Chan K.W. (2016). Polarization of Tissue-Resident TFH-Like Cells in Human Hepatoma Bridges Innate Monocyte Inflammation and M2b Macrophage Polarization. Cancer Discov..

[234] Wu H., Tang S., Zhou M., Xue J., Yu Z., Zhu J. (2021). Tim-3 suppresses autoimmune hepatitis via the p38/MKP-1 pathway in Th17 cells. FEBS Open Bio..

[235] Schatton T., Itoh Y., Martins C., Rasbach E., Singh P., Silva M., Mucciarone K., Heppt M.V., Geddes-Sweeney J., Stewart K. (2022). Inhibition of Melanoma Cell-Intrinsic Tim-3 Stimulates MAPK-Dependent Tumorigenesis. Cancer Res..

[236] Lee C.S., Yuan T.L., Chakka S., Fellmann C., Lowe S.W., Caplen N.J., McCormick F., Luo J. (2019). MAP kinase and autophagy pathways cooperate to maintain RAS mutant cancer cell survival. Proc. Natl. Acad. Sci. USA.

[237] Fuhrman C.A., Yeh W.I., Seay H.R., Saikumar Lakshmi P., Chopra G., Zhang L., Perry D.J., McClymont S.A., Yadav M., Lopez M.C. (2015). Divergent Phenotypes of Human Regulatory T Cells Expressing the Receptors TIGIT and CD226. J. Immunol..

[238] Wang N., Liang S., Jin J., Fang L., Ma Q., Wang X., Song Y. (2019). Chen LCD226 attenuates Treg suppressive capacity via, C.T.L.A.-4.; TIGIT during, E.A.E. Immunol. Res..

[239] Zhang J., Zhou L., Xiang J.D., Jin C.S., Li M.Q., He Y.Y. (2021). Artesunate-induced ATG5-related autophagy enhances the cytotoxicity of NK92 cells on endometrial cancer cells via interactions between CD155 and CD226/TIGIT. Int. Immunopharmacol..

[240] Reches A., Ophir Y., Stein N., Kol I., Isaacson B., Charpak-Amikam Y., Elnekave A., Tsukerman P., Kucan Brlic P., Lenac T. (2020). Nectin4 is a Novel TIGIT Ligand Which Combines Checkpoint Inhibition and Tumor Specificity. J. Immunother. Cancer.

[241] Yang B., Xue Q., Guo J., Wang X., Zhang Y., Guo K., Li W., Chen S., Xue T., Qi X. (2020). Autophagy induction by the pathogen receptor NECTIN4 and sustained autophagy contribute to peste des petits ruminants virus infectivity. Autophagy.

[242] Caramalho Í., Nunes-Cabaço H., Foxall R.B., Sousa A.E. (2015). Regulatory T-Cell Development in the Human Thymus. Front. Immunol..

[243] Richards D.M., Delacher M., Goldfarb Y., Kägebein D., Hofer A.C., Abramson J., Feuerer M. (2015). Treg Cell Differentiation: From Thymus to Peripheral Tissue. Prog. Mol. Biol. Transl. Sci..

[244] Ryba-Stanisławowska M., Sakowska J., Zieliński M., Ławrynowicz U., Trzonkowski P. (2019). Regulatory T cells: The future of autoimmune disease treatment. Expert Rev. Clin. Immunol..

[245] Maceiras A.R., Almeida S.C.P., Mariotti-Ferrandiz E., Chaara W., Jebbawi F., Six A., Hori S., Klatzmann D., Faro J., Graca L. (2017). T follicular helper and T follicular regulatory cells have different TCR specificity. Nat. Commun..

[246] Vaeth M., Müller G., Stauss D., Dietz L., Klein-Hessling S., Serfling E., Lipp M., Berberich I., Berberich-Siebelt F. (2014). Follicular regulatory T cells control humoral autoimmunity via NFAT2-regulated CXCR5 expression. J. Exp. Med..

[247] Fisson S., Darrasse-Jèze G., Litvinova E., Septier F., Klatzmann D., Liblau R., Salomon B.L. (2003). Continuous activation of autoreactive CD4+ CD25+ regulatory T cells in the steady state. J. Exp. Med..

[248] Lewkowicz N., Klink M., Mycko M.P., Lewkowicz P. (2013). Neutrophil-CD4+CD25+ T regulatory cell interactions: A possible new mechanism of infectious tolerance. Immunobiology.

[249] Piekarska K., Urban-Wójciuk Z., Kurkowiak M., Pelikant-Małecka I., Schumacher A., Sakowska J., Spodnik J.H., Arcimowicz Ł., Zielińska H., Tymoniuk B. (2022). Mesenchymal stem cells transfer mitochondria to allogeneic Tregs in an HLA-dependent manner improving their immunosuppressive activity. Nat. Commun..

[250] Robbins P.D., Morelli A.E. (2014). Regulation of immune responses by extracellular vesicles. Nat. Rev. Immunol..

[251] Sullivan J.A., Tomita Y., Jankowska-Gan E., Lema D.A., Arvedson M.P., Nair A., Bracamonte-Baran W., Zhou Y., Meyer K.K., Zhong W. (2020). Treg-Cell-Derived IL-35-Coated Extracellular Vesicles Promote Infectious Tolerance. Cell Rep..

[252] Barzaghi F., Passerini L. (2021). IPEX Syndrome: Improved Knowledge of Immune Pathogenesis Empowers Diagnosis. Front. Pediatr..

[253] Wildin R.S., Ramsdell F., Peake J., Faravelli F., Casanova J.L., Buist N., Levy-Lahad E., Mazzella M., Goulet O., Perroni L. (2001). X-linked neonatal diabetes mellitus, enteropathy and endocrinopathy syndrome is the human equivalent of mouse scurfy. Nat. Genet..

[254] de Kleer I.M., Wedderburn L.R., Taams L.S., Patel A., Varsani H., Klein M., de Jager W., Pugayung G., Giannoni F., Rijkers G. (2004). CD4+CD25bright regulatory T cells actively regulate inflammation in the joints of patients with the remitting form of juvenile idiopathic arthritis. J. Immunol..

[255] van Amelsfort J.M., Jacobs K.M., Bijlsma J.W., Lafeber F.P., Taams L.S. (2004). CD4(+)CD25(+) regulatory T cells in rheumatoid arthritis: Differences in the presence, phenotype, and function between peripheral blood and synovial fluid. Arthritis Rheum..

[256] Slobodin G., Ahmad M.S., Rosner I., Peri R., Rozenbaum M., Kessel A., Toubi E., Odeh M. (2010). Regulatory T cells (CD4(+)CD25(bright)FoxP3(+)) expansion in systemic sclerosis correlates with disease activity and severity. Cell Immunol..

[257] La Cava A. (2018). Tregs in SLE: An Update. Curr. Rheumatol. Rep..

[258] Vitales-Noyola M., Serrano-Somavilla A., Martínez-Hernández R., Sampedro-Nuñez M., Ramos-Levi A.M., González-Amaro R., Marazuela M. (2018). Patients with Autoimmune Thyroiditis Show Diminished Levels and Defective Suppressive Function of Tr1 Regulatory Lymphocytes. J. Clin. Endocrinol. Metab..

[259] Marazuela M., García-López M.A., Figueroa-Vega N., De La Fuente H., Alvarado B., Monsiváis-Urenda A., Sánchez-Madrid F., González-Amaro R. (2006). Regulatory T Cells in Human Autoimmune Thyroid Disease. J. Clin. Endocrinol. Metab..

[260] Kumar M., Putzki N., Limmroth V., Remus R., Lindemann M., Knop D., Mueller N., Hardt C., Kreuzfelder E., Grosse-Wilde H. (2006). CD4+CD25+FoxP3+ T lymphocytes fail to suppress myelin basic protein-induced proliferation in patients with multiple sclerosis. J. Neuroimmunol..

[261] Venken K., Hellings N., Thewissen M., Somers V., Hensen K., Rummens J.L., Medaer R., Hupperts R., Stinissen P. (2008). Compromised CD4+ CD25(high) regulatory T-cell function in patients with relapsing-remitting multiple sclerosis is correlated with a reduced frequency of FOXP3-positive cells and reduced FOXP3 expression at the single-cell level. Immunology.

[262] Holohan D.R., Van Gool F., Bluestone J.A. (2019). Thymically-derived Foxp3+ regulatory T cells are the primary regulators of type 1 diabetes in the non-obese diabetic mouse model. PLoS ONE.

[263] Bending D., Giannakopoulou E., Lom H., Wedderburn L.R. (2015). Synovial Regulatory T Cells Occupy a Discrete TCR Niche in Human Arthritis and Require Local Signals To Stabilize FOXP3 Protein Expression. J. Immunol..

[264] Yang S., Zhang X., Chen J., Dang J., Liang R., Zeng D., Zhang H., Xue Y., Liu Y., Wu W. (2020). Induced, But Not Natural, Regulatory T Cells Retain Phenotype and Function Following Exposure to Inflamed Synovial Fibroblasts. Sci. Adv..

[265] Pesenacker A.M., Bending D., Ursu S., Wu Q., Nistala K., Wedderburn L. (2013). CD161 defines the subset of FoxP3+ T cells capable of producing proinflammatory cytokines. Blood.

[266] Qiu R., Zhou L., Ma Y., Zhou L., Liang T., Shi L., Long J., Yuan D. (2020). Regulatory T Cell Plasticity and Stability and Autoimmune Diseases. Clin. Rev. Allergy Immunol..

[267] Yang X.O., Nurieva R., Martinez G.J., Kang H.S., Chung Y., Pappu B.P., Shah B., Chang S.H., Schluns K.S., Watowich S.S. (2008). Molecular antagonism and plasticity of regulatory and inflammatory T cell programs. Immunity.

[268] Zhang J., Chen L., Xiong F., Zhang S., Huang K., Zhang Z., Wang C.Y. (2019). Autophagy in regulatory T cells: A double-edged sword in disease settings. Mol. Immunol..

[269] Alissafi T., Banos A., Boon L., Sparwasser T., Ghigo A., Wing K., Vassilopoulos D., Boumpas D., Chavakis T., Cadwell K. (2017). Tregs restrain dendritic cell autophagy to ameliorate autoimmunity. J. Clin. Invest..

[270] de Oliveira C.E., Gasparoto T.H., Pinheiro C.R., Amôr N.G., Nogueira M.R.S., Kaneno R., Garlet G.P., Lara V.S., Silva J.S., Cavassani K.A. (2017). CCR5-Dependent Homing of T Regulatory Cells to the Tumor Microenvironment Contributes to Skin Squamous Cell Carcinoma Development. Mol. Cancer Ther..

[271] Mizukami Y., Kono K., Kawaguchi Y., Akaike H., Kamimura K., Sugai H., Fujii H. (2008). CCL17 and CCL22 chemokines within tumor microenvironment are related to accumulation of Foxp3+ regulatory T cells in gastric cancer. Int. J. Cancer..

[272] Lu L., Ma J., Li Z., Lan Q., Chen M., Liu Y., Xia Z., Wang J., Han Y., Shi W. (2011). All-trans retinoic acid promotes TGF-β-induced Tregs via histone modification but not DNA demethylation on Foxp3 gene locus. PLoS ONE.

[273] Shitara K., Nishikawa H. (2018). Regulatory T cells: A potential target in cancer immunotherapy. Ann. N. York Acad. Sci..

[274] Chaudhary B., Elkord E. (2016). Regulatory T Cells in the Tumor Microenvironment and Cancer Progression: Role and Therapeutic Targeting. Vaccines.

[275] Que W., Guo W.Z., Li X.K. (2020). Manipulation of Regulatory Dendritic Cells for Induction Transplantation Tolerance. Front. Immunol..

[276] Lazarova M., Steinle A. (2019). Impairment of NKG2D-Mediated Tumor Immunity by TGF-β. Front. Immunol..

[277] Lindqvist C.A., Christiansson L.H., Simonsson B., Enblad G., Olsson-Strömberg U., Loskog A.S. (2010). T regulatory cells control T-cell proliferation partly by the release of soluble CD25 in patients with B-cell malignancies. Immunology.

[278] Sawant D.V., Yano H., Chikina M., Zhang Q., Liao M., Liu C., Callahan D.J., Sun Z., Sun T., Tabib T. (2019). Adaptive plasticity of IL-10+ and IL-35+ Treg cells cooperatively promotes tumor T cell exhaustion. Nat. Immunol..

[279] Turnis M.E., Sawant D.V., Szymczak-Workman A.L., Andrews L.P., Delgoffe G.M., Yano H., Beres A.J., Vogel P., Workman C.J., Vignali D.A. (2016). Interleukin-35 Limits Anti-Tumor Immunity. Immunity.

[280] Correale P., Rotundo M.S., Del Vecchio M.T., Remondo C., Migali C., Ginanneschi C., Tsang K.Y., Licchetta A., Mannucci S., Loiacono L. (2010). Regulatory (FoxP3+) T-cell tumor infiltration is a favorable prognostic factor in advanced colon cancer patients undergoing chemo or chemoimmunotherapy. J. Immunother..

[281] Shen Z., Zhou S., Wang Y., Li R.L., Zhong C., Liang C., Sun Y. (2010). Higher intratumoral infiltrated Foxp3+ Treg numbers and Foxp3+/CD8+ ratio are associated with adverse prognosis in resectable gastric cancer. J. Cancer Res. Clin. Oncol..

[282] Bonertz A., Weitz J., Pietsch D.H., Rahbari N.N., Schlude C., Ge Y., Juenger S., Vlodavsky I., Khazaie K., Jaeger D. (2009). Antigen-specific Tregs control T cell responses against a limited repertoire of tumor antigens in patients with colorectal carcinoma. J. Clin. Investig..

[283] Wei J., Long L., Yang K., Guy C., Shrestha S., Chen Z., Wu C., Vogel P., Neale G., Green D.R. (2016). Autophagy enforces functional integrity of regulatory T cells by coupling environmental cues and metabolic homeostasis. Nat. Immunol..

[284] Le Texier L., Lineburg K.E., Cao B., McDonald-Hyman C., Leveque-El Mouttie L., Nicholls J., Melino M., Nalkurthi B.C., Alexander K.A., Teal B. (2016). Autophagy-dependent regulatory T cells are critical for the control of graft-versus-host disease. JCI Insight.

[285] Duong B.H., Ota T., Aoki-Ota M., Cooper A.B., Ait-Azzouzene D., Vela J.L., Gavin A.L., Nemazee D. (2011). Negative selection by IgM superantigen defines a B cell central tolerance compartment and reveals mutations allowing escape. J. Immunol..

[286] Nemazee D. (2017). Mechanisms of central tolerance for B cells. Nat. Rev. Immunol..

[287] Zhao Y., Shen M., Feng Y., He R., Xu X., Xie Y., Shi X., Zhou M., Pan S., Wang M. (2017). Regulatory B cells induced by pancreatic cancer cell-derived interleukin-18 promote immune tolerance via the PD-1/PD-L1 pathway. Oncotarget.

[288] Ding Q., Yeung M., Camirand G., Zeng Q., Akiba H., Yagita H., Chalasani G., Sayegh M.H., Najafian N., Rothstein D.M. (2011). Regulatory B cells are identified by expression of TIM-1 and can be induced through TIM-1 ligation to promote tolerance in mice. J. Clin. Invest..

[289] Baba Y., Saito Y., Kotetsu Y. (2020). Heterogeneous subsets of B-lineage regulatory cells (Breg cells). Int. Immunol..

[290] Chen Y., Li C., Lu Y., Zhuang H., Gu W., Liu B., Liu F., Sun J., Yan B., Weng D. (2017). IL-10-Producing CD1dhiCD5+ Regulatory B Cells May Play a Critical Role in Modulating Immune Homeostasis in Silicosis Patients. Front. Immunol..

[291] Noh J., Lee J.H., Noh G., Bang S.Y., Kim H.S., Choi W.S., Cho S., Lee S.S. (2010). Characterisation of allergen-specific responses of IL-10-producing regulatory B cells (Br1) in Cow Milk Allergy. Cell Immunol..

[292] Iwata Y., Matsushita T., Horikawa M., Dilillo D.J., Yanaba K., Venturi G.M., Szabolcs P.M., Bernstein S.H., Magro C.M., Williams A.D. (2011). Characterization of a rare IL-10-competent B-cell subset in humans that parallels mouse regulatory B10 cells. Blood.

[293] Blair P.A., Noreña L.Y., Flores-Borja F., Rawlings D.J., Isenberg D.A., Ehrenstein M.R., Mauri C. (2010). CD19(+)CD24(hi)CD38(hi) B cells exhibit regulatory capacity in healthy individuals but are functionally impaired in systemic Lupus Erythematosus patients. Immunity.

[294] Wang W., Yuan X., Chen H., Xie G., Ma Y., Zheng Y., Zhou Y., Shen L. (2015). CD19+CD24hiCD38hiBregs involved in downregulate helper T cells and upregulate regulatory T cells in gastric cancer. Oncotarget.

[295] Flores-Borja F., Bosma A., Ng D., Reddy V., Ehrenstein M.R., Isenberg D.A., Mauri C. (2013). CD19+CD24hiCD38hi B cells maintain regulatory T cells while limiting TH1 and TH17 differentiation. Sci. Transl. Med..

[296] van de Veen W., Stanic B., Yaman G., Wawrzyniak M., Söllner S., Akdis D.G., Rückert B., Akdis C.A., Akdis M. (2013). IgG4 production is confined to human IL-10-producing regulatory B cells that suppress antigen-specific immune responses. J. Allergy Clin. Immunol..

[297] Díaz-Alderete A., Crispin J.C., Vargas-Rojas M.I., Alcocer-Varela J. (2004). IL-10 production in B cells is confined to CD154+ cells in patients with systemic lupus erythematosus. J. Autoimmun..

[298] Xiao X., Lao X.-M., Chen M.-M., Liu R.-X., Wei Y., Ouyang F.-Z., Chen D.-P., Zhao X.-Y., Zhao Q., Li X.-F. (2016). PD-1hi Identifies a Novel Regulatory B-cell Population in Human Hepatoma That Promotes Disease Progression. Cancer Discov..

[299] Matsumoto M., Baba A., Yokota T., Nishikawa H., Ohkawa Y., Kayama H., Kallies A., Nutt S.L., Sakaguchi S., Takeda K. (2014). Interleukin-10-producing plasmablasts exert regulatory function in autoimmune inflammation. Immunity.

[300] Kessel A., Haj T., Peri R., Snir A., Melamed D., Sabo E., Toubi E. (2012). Human CD19(+)CD25(high) B regulatory cells suppress proliferation of CD4(+) T cells and enhance Foxp3 and CTLA-4 expression in T-regulatory cells. Autoimmun. Rev..

[301] Matsushita T. (2019). Regulatory and effector B cells: Friends or foes?. J. Dermatol. Sci..

[302] Yoshizaki A., Miyagaki T., DiLillo D.J., Matsushita T., Horikawa M., Kountikov E.I., Spolski R., Poe J.C., Leonard W.J., Tedder T.F. (2012). Regulatory B cells control T-cell autoimmunity through IL-21-dependent cognate interactions. Nature.

[303] Mauri C., Gray D., Mushtaq N., Londei M. (2003). Prevention of arthritis by interleukin 10-producing B cells. J. Exp. Med..

[304] Watanabe R., Ishiura N., Nakashima H., Kuwano Y., Okochi H., Tamaki K., Sato S., Tedder T.F., Fujimoto M. (2010). Regulatory B cells (B10 cells) have a suppressive role in murine lupus: CD19 and B10 cell deficiency exacerbates systemic autoimmunity. J. Immunol..

[305] Matsushita T., Yanaba K., Bouaziz J.D., Fujimoto M., Tedder T.F. (2008). Regulatory B cells inhibit EAE initiation in mice while other B cells promote disease progression. J. Clin. Investig..

[306] Lo-Man R. (2011). Regulatory B cells control dendritic cell functions. Immunotherapy.

[307] Yang M., Rui K., Wang S., Lu L. (2013). Regulatory B cells in autoimmune diseases. Cell Mol. Immunol..

[308] Yuen G.J., Demissie E., Pillai S. (2016). B lymphocytes and cancer: A love-hate relationship. Trends Cancer.

[309] Zhou J., Min Z., Zhang D., Wang W., Marincola F., Wang X. (2014). Enhanced frequency and potential mechanism of B regulatory cells in patients with lung cancer. J. Transl. Med..

[310] Murakami Y., Saito H., Shimizu S., Kono Y., Shishido Y., Miyatani K., Matsunaga T., Fukumoto Y., Ashida K., Sakabe T. (2019). Increased regulatory B cells are involved in immune evasion in patients with gastric cancer. Sci. Rep..

[311] Ishigami E., Sakakibara M., Sakakibara J., Masuda T., Fujimoto H., Hayama S., Nagashima T., Sangai T., Nakagawa A., Nakatani Y. (2019). Coexistence of regulatory B cells and regulatory T cells in tumor-infiltrating lymphocyte aggregates is a prognostic factor in patients with breast cancer. Breast Cancer.

[312] Lizotte P.H., Ivanova E.V., Awad M.M., Jones R.E., Keogh L., Liu H., Dries R., Almonte C., Herter-Sprie G.S., Santos A. (2016). Multiparametric profiling of non-small-cell lung cancers reveals distinct immunophenotypes. JCI Insight..

[313] Zhang Y., Morgan R., Chen C., Cai Y., Clark E., Khan W.N., Shin S.U., Cho H.M., Al Bayati A., Pimentel A. (2016). Mammary-tumor-educated B cells acquire LAP/TGF-β and PD-L1 expression and suppress anti-tumor immune responses. Int. Immunol..

[314] Lindner S., Dahlke K., Sontheimer K., Hagn M., Kaltenmeier C., Barth T.F., Beyer T., Reister F., Fabricius D., Lotfi R. (2013). Interleukin 21-induced granzyme B-expressing B cells infiltrate tumors and regulate T cells. Cancer Res..

[315] Bodogai M., Moritoh K., Lee-Chang C., Hollander C.M., Sherman-Baust C.A., Wersto R.P., Araki Y., Miyoshi I., Yang L., Trinchieri G. (2015). Immunosuppressive and Prometastatic Functions of Myeloid-Derived Suppressive Cells Rely upon Education from Tumor-Associated B Cells. Cancer Res..

[316] Olkhanud P.B., Damdinsuren B., Bodogai M., Gress R.E., Sen R., Wejksza K., Malchinkhuu E., Wersto R.P., Biragyn A. (2011). Tumor-evoked regulatory B cells promote breast cancer metastasis by converting resting CD4⁺ T cells to T-regulatory cells. Cancer Res..

[317] Pylayeva-Gupta Y., Das S., Handler J.S., Hajdu C.H., Coffre M., Koralov S.B., Bar-Sagi D. (2016). IL35-Producing B Cells Promote the Development of Pancreatic Neoplasia. Cancer Discov..

[318] Zhou M., Wen Z., Cheng F., Ma J., Li W., Ren H., Sheng Y., Dong H., Lu L., Hu H.M. (2016). Tumor-released autophagosomes induce IL-10-producing B cells with suppressive activity on T lymphocytes via TLR2-MyD88-NF-κB signal pathway. Oncoimmunology.

[319] Corsale A.M., Di Simone M., Lo Presti E., Dieli F., Meraviglia S. (2023). γδ T cells and their clinical application in colon cancer. Front. Immunol..

[320] Nielsen M.M., Witherden D.A., Havran W.L. (2017). γδ T cells in homeostasis and host defence of epithelial barrier tissues. Nat. Rev Immunol..

[321] Mikulak J., Oriolo F., Bruni E., Roberto A., Colombo F.S., Villa A., Bosticardo M., Bortolomai I., Presti E.L., Meraviglia S. (2019). NKp46-expressing human gut-resident intraepithelial Vδ1 T cell subpopulation exhibits high antitumor activity against colorectal cancer. JCI Insight.

[322] Shiromizu C.M., Jancic C.C. (2018). γδ T Lymphocytes: An Effector Cell in Autoimmunity and Infection. Front. Immunol..

[323] Isailovic N., Daigo K., Mantovani A., Selmi C. (2015). Interleukin-17 and innate immunity in infections and chronic inflammation. J. Autoimmun..

[324] Burkett P.R., Meyer zu Horste G., Kuchroo V.K. (2015). Pouring fuel on the fire: Th17 cells, the environment, and autoimmunity. J. Clin. Investig..

[325] Papotto P.H., Reinhardt A., Prinz I., Silva-Santos B. (2018). Innately versatile: γδ17 T cells in inflammatory and autoimmune diseases. J. Autoimmun..

[326] Moens E., Brouwer M., Dimova T., Goldman M., Willems F., Vermijlen D. (2011). IL-23R and TCR signaling drives the generation of neonatal Vgamma9Vdelta2 T cells expressing high levels of cytotoxic mediators and producing IFN-gamma and IL-17. J. Leukoc Biol..

[327] Doherty D.G. (2016). Immunity, tolerance and autoimmunity in the liver: A comprehensive review. J. Autoimmun..

[328] Cong L.H., Li T., Wang H., Wu Y.N., Wang S.P., Zhao Y.Y., Zhang G.Q., Duan J. (2020). IL-17A-producing T cells exacerbate fine particulate matter-induced lung inflammation and fibrosis by inhibiting PI3K/Akt/mTOR-mediated autophagy. J. Cell Mol. Med..

[329] Wang Z., Zhou H., Zheng H., Zhou X., Shen G., Teng X., Liu X., Zhang J., Wei X., Hu Z. (2021). Autophagy-based unconventional secretion of HMGB1 by keratinocytes plays a pivotal role in psoriatic skin inflammation. Autophagy.

[330] Silva-Santos B., Serre K., Norell H. (2015). γδ T cells in cancer. Nat. Rev. Immunol..

[331] Gao Z., Bai Y., Lin A., Jiang A., Zhou C., Cheng Q., Liu Z., Chen X., Zhang J., Luo P. (2023). Gamma delta T-cell-based immune checkpoint therapy: Attractive candidate for antitumor treatment. Mol. Cancer.

[332] Liu Y., Zhang C. (2020). The Role of Human γδ T Cells in Anti-Tumor Immunity and Their Potential for Cancer Immunotherapy. Cells.

[333] Tao L.F., Yang B.Q., Zeng Z.Y., Xu J.P., Lin D.H., Chen Q.C., Chen J.M. (2022). Effect of γδ T cells on the Proliferation, Apoptosis and Autophagy of Multiple Myeloma Cells. Zhongguo Shi Yan Xue Ye Xue Za Zhi.

[334] Wu P., Wu D., Ni C., Ye J., Chen W., Hu G., Wang Z., Wang C., Zhang Z., Xia W. (2014). γδT17 cells promote the accumulation and expansion of myeloid-derived suppressor cells in human colorectal cancer. Immunity.

[335] Meraviglia S., Lo Presti E., Tosolini M., La Mendola C., Orlando V., Todaro M., Catalano V., Stassi G., Cicero G., Vieni S. (2017). Distinctive features of tumor-infiltrating γδ T lymphocytes in human colorectal cancer. Oncoimmunology.

[336] Bronte V., Brandau S., Chen S.H., Colombo M.P., Frey A.B., Greten T.F., Mandruzzato S., Murray P.J., Ochoa A., Ostrand-Rosenberg S. (2016). Recommendations for myeloid-derived suppressor cell nomenclature and characterization standards. Nat. Commun..

[337] Sipos F., Műzes G. (2023). Cancer Stem Cell Relationship with Pro-Tumoral Inflammatory Microenvironment. Biomedicines.

[338] Li C., Liu T., Bazhin A.V., Yang Y. (2017). The Sabotaging Role of Myeloid Cells in Anti-Angiogenic Therapy: Coordination of Angiogenesis and Immune Suppression by Hypoxia. J. Cell Physiol..

[339] Toh B., Wang X., Keeble J., Sim W.J., Khoo K., Wong W.C., Kato M., Prevost-Blondel A., Thiery J.P., Abastado J.P. (2011). Mesenchymal transition and dissemination of cancer cells is driven by myeloid-derived suppressor cells infiltrating the primary tumor. PLoS Biol..

[340] Raber P.L., Thevenot P., Sierra R., Wyczechowska D., Halle D., Ramirez M.E., Ochoa A.C., Fletcher M., Velasco C., Wilk A. (2014). Subpopulations of myeloid-derived suppressor cells impair T cell responses through independent nitric oxide-related pathways. Int. J. Cancer.

[341] Markowitz J., Wang J., Vangundy Z., You J., Yildiz V., Yu L., Foote I.P., Branson O.E., Stiff A.R., Brooks T.R. (2017). Nitric oxide mediated inhibition of antigen presentation from DCs to CD4+ T cells in cancer and measurement of STAT1 nitration. Sci. Rep..

[342] Kusmartsev S., Nefedova Y., Yoder D., Gabrilovich D.I. (2004). Antigen-specific inhibition of CD8+ T cell response by immature myeloid cells in cancer is mediated by reactive oxygen species. J. Immunol..

[343] Yang Y., Bazhin A.V., Werner J., Karakhanova S. (2013). Reactive oxygen species in the immune system. Int. Rev. Immunol..

[344] Chen X., Song M., Zhang B., Zhang Y. (2016). Reactive Oxygen Species Regulate T Cell Immune Response in the Tumor Microenvironment. Oxid. Med. Cell Longev..

[345] Rodríguez P.C., Ochoa A.C. (2008). Arginine regulation by myeloid derived suppressor cells and tolerance in cancer: Mechanisms and therapeutic perspectives. Immunol. Rev..

[346] Srivastava M.K., Sinha P., Clements V.K., Rodriguez P., Ostrand-Rosenberg S. (2010). Myeloid-derived suppressor cells inhibit T-cell activation by depleting cystine and cysteine. Cancer Res..

[347] Baniyash M. (2004). TCR zeta-chain downregulation: Curtailing an excessive inflammatory immune response. Nat. Rev. Immunol..

[348] Yu J., Du W., Yan F., Wang Y., Li H., Cao S., Yu W., Shen C., Liu J., Ren X. (2013). Myeloid-derived suppressor cells suppress antitumor immune responses through IDO expression and correlate with lymph node metastasis in patients with breast cancer. J. Immunol..

[349] Platten M., Wick W., Van den Eynde B.J. (2012). Tryptophan catabolism in cancer: Beyond IDO and tryptophan depletion. Cancer Res..

[350] Munn D.H., Sharma M.D., Baban B., Harding H.P., Zhang Y., Ron D., Mellor A.L. (2005). GCN2 kinase in T cells mediates proliferative arrest and anergy induction in response to indoleamine 2,3-dioxygenase. Immunity.

[351] Nagaraj S., Gupta K., Pisarev V., Kinarsky L., Sherman S., Kang L., Herber D.L., Schneck J., Gabrilovich D.I. (2007). Altered recognition of antigen is a mechanism of CD8+ T cell tolerance in cancer. Nat. Med..

[352] Bronte V., Kasic T., Gri G., Gallana K., Borsellino G., Marigo I., Battistini L., Iafrate M., Prayer-Galetti T., Pagano F. (2005). Boosting antitumor responses of T lymphocytes infiltrating human prostate cancers. J. Exp. Med..

[353] Lu T., Gabrilovich D.I. (2012). Molecular pathways: Tumor-infiltrating myeloid cells and reactive oxygen species in regulation of tumor microenvironment. Clin. Cancer Res..

[354] Li J., Wang L., Chen X., Li L., Li Y., Ping Y., Huang L., Yue D., Zhang Z., Wang F. (2017). CD39/CD73 upregulation on myeloid-derived suppressor cells via TGF-β-mTOR-HIF-1 signaling in patients with non-small cell lung cancer. Oncoimmunology.

[355] Li L., Wang L., Li J., Fan Z., Yang L., Zhang Z., Zhang C., Yue D., Qin G., Zhang T. (2018). Metformin-Induced Reduction of CD39 and CD73 Blocks Myeloid-Derived Suppressor Cell Activity in Patients with Ovarian Cancer. Cancer Res..

[356] Umansky V., Shevchenko I., Bazhin A.V., Utikal J. (2014). Extracellular adenosine metabolism in immune cells in melanoma. Cancer Immunol. Immunother..

[357] Hu C.E., Gan J., Zhang R.D., Cheng Y.R., Huang G.J. (2011). Up-regulated myeloid-derived suppressor cell contributes to hepatocellular carcinoma development by impairing dendritic cell function. Scand. J. Gastroenterol..

[358] Li H., Han Y., Guo Q., Zhang M., Cao X. (2009). Cancer-expanded myeloid-derived suppressor cells induce anergy of NK cells through membrane-bound TGF-beta 1. J. Immunol..

[359] Ghiringhelli F., Puig P.E., Roux S., Parcellier A., Schmitt E., Solary E., Kroemer G., Martin F., Chauffert B., Zitvogel L. (2005). Tumor cells convert immature myeloid dendritic cells into TGF-beta-secreting cells inducing CD4+CD25+ regulatory T cell proliferation. J. Exp. Med..

[360] Zhang F., Wang H., Wang X., Jiang G., Liu H., Zhang G., Wang H., Fang R., Bu X., Cai S. (2016). TGF-β induces M2-like macrophage polarization via SNAIL-mediated suppression of a pro-inflammatory phenotype. Oncotarget.

[361] Fridlender Z.G., Sun J., Kim S., Kapoor V., Cheng G., Ling L., Worthen G.S., Albelda S.M. (2009). Polarization of tumor-associated neutrophil phenotype by TGF-beta: “N1” versus “N2” TAN. Cancer Cell.

[362] Xu T., Jiang L., Wang Z. (2018). The progression of HMGB1-induced autophagy in cancer biology. Onco. Targets Ther..

[363] Alissafi T., Hatzioannou A., Mintzas K., Barouni R.M., Banos A., Sormendi S., Polyzos A., Xilouri M., Wielockx B., Gogas H. (2018). Autophagy orchestrates the regulatory program of tumor-associated myeloid-derived suppressor cells. J. Clin. Investig..

[364] Shibata M., Nanno K., Yoshimori D., Nakajima T., Takada M., Yazawa T., Mimura K., Inoue N., Watanabe T., Tachibana K. (2022). Myeloid-derived suppressor cells: Cancer, autoimmune diseases, and more. Oncotarget.

[365] Moliné-Velázquez V., Cuervo H., Vila-Del Sol V., Ortega M.C., Clemente D., De Castro F. (2011). Myeloid-Derived Suppressor Cells Limit the Inflammation by Promoting T Lymphocyte Apoptosis in the Spinal Cord of a Murine Model of Multiple Sclerosis. Brain Pathol..

[366] Highfill S.L., Rodriguez P.C., Zhou Q., Goetz C.A., Koehn B.H., Veenstra R., Taylor P.A., Panoskaltsis-Mortari A., Serody J.S., Munn D.H. (2010). Bone Marrow Myeloid-Derived Suppressor Cells (MDSCs) Inhibit Graft-Versus-Host Disease (GVHD) via an Arginase-1-Dependent Mechanism That is Up-Regulated by Interleukin-13. Blood.

[367] Ioannou M., Alissafi T., Lazaridis I., Deraos G., Matsoukas J., Gravanis A., Mastorodemos V., Plaitakis A., Sharpe A., Boumpas D. (2012). Crucial role of granulocytic myeloid-derived suppressor cells in the regulation of central nervous system autoimmune disease. J. Immunol..

[368] Veglia F., Sanseviero E., Gabrilovich D.I. (2021). Myeloid-derived suppressor cells in the era of increasing myeloid cell diversity. Nat. Rev. Immunol..

[369] Guo C., Hu F., Yi H., Feng Z., Li C., Shi L., Li Y., Liu H., Yu X., Wang H. (2016). Myeloid-derived suppressor cells have a proinflammatory role in the pathogenesis of autoimmune arthritis. Ann. Rheum. Dis..

[370] Wang Z., Zhu F., Wang J., Tao Q., Xu X., Wang H., Xiong S., Wang Y., Zhai Z. (2019). Increased CD14+HLA-DR-/low Myeloid-Derived Suppressor Cells Correlate With Disease Severity in Systemic Lupus Erythematosus Patients in an iNOS-Dependent Manner. Front. Immunol..

[371] Jiao Z., Hua S., Wang W., Wang H., Gao J., Wang X. (2013). Increased circulating myeloid-derived suppressor cells correlated negatively with Th17 cells in patients with rheumatoid arthritis. Scand. J. Rheumatol..

[372] Yin B., Ma G., Yen C.-Y., Zhou Z., Wang G.X., Divino C.M., Casares S., Chen S.-H., Yang W.-C., Pan P.-Y. (2010). Myeloid-Derived Suppressor Cells Prevent Type 1 Diabetes in Murine Models. J. Immunol..

[373] Dong G., Si C., Zhang Q., Yan F., Li C., Zhang H., Ma Q., Dai J., Li Z., Shi H. (2017). Autophagy regulates accumulation and functional activity of granulocytic myeloid-derived suppressor cells via STAT3 signaling in endotoxin shock. Biochim. Biophys. Acta Mol. Basis. Dis..

[374] Kotze L.A., Leukes V.N., Fang Z., Lutz M.B., Fitzgerald B.L., Belisle J., Loxton A.G., Walzl G., du Plessis N. (2021). Evaluation of autophagy mediators in myeloid-derived suppressor cells during human tuberculosis. Cell Immunol..

[375] Shapouri-Moghaddam A., Mohammadian S., Vazini H., Taghadosi M., Esmaeili S.A., Mardani F., Seifi B., Mohammadi A., Afshari J.T., Sahebkar A. (2018). Macrophage plasticity, polarization, and function in health and disease. J. Cell Physiol..

[376] Pires-Afonso Y., Niclou S.P., Michelucci A. (2020). Revealing and Harnessing Tumour-Associated Microglia/Macrophage Heterogeneity in Glioblastoma. Int. J. Mol. Sci..

[377] Ueno T., Toi M., Saji H., Muta M., Bando H., Kuroi K., Koike M., Inadera H., Matsushima K. (2000). Significance of macrophage chemoattractant protein-1 in macrophage recruitment, angiogenesis, and survival in human breast cancer. Clin. Cancer Res..

[378] Jin L., Guo Y., Mao W., Wang J., Jin L., Liu X., Shou Q., Fu H. (2022). Total glucosides of paeony inhibit breast cancer growth by inhibiting TAMs infiltration through NF-κB/CCL2 signaling. Phytomedicine.

[379] Shanmugam G., Das S., Paul S., Rakshit S., Sarkar K. (2022). Clinical relevance and therapeutic aspects of professional antigen-presenting cells in lung cancer. Med. Oncol..

[380] McClellan J.L., Davis J.M., Steiner J.L., Enos R.T., Jung S.H., Carson J.A., Pena M.M., Carnevale K.A., Berger F.G., Murphy E.A. (2012). Linking tumor-associated macrophages, inflammation, and intestinal tumorigenesis: Role of MCP-1. Am. J. Physiol. Liver Physiol..

[381] Rhee I. (2016). Diverse macrophages polarization in tumor microenvironment. Arch. Pharm. Res..

[382] Yuen K.C., Liu L.F., Gupta V., Madireddi S., Keerthivasan S., Li C., Rishipathak D., Williams P., Kadel E.E., Koeppen H. (2020). High systemic and tumor-associated IL-8 correlates with reduced clinical benefit of PD-L1 blockade. Nat. Med..

[383] Mittal S.K., Roche P.A. (2015). Suppression of antigen presentation by IL-10. Curr. Opin. Immunol..

[384] Luo X., Qiu Y., Dinesh P., Gong W., Jiang L., Feng X., Li J., Jiang Y., Lei Y.L., Chen Q. (2021). The functions of autophagy at the tumour-immune interface. J. Cell Mol. Med..

[385] Germic N., Frangez Z., Yousefi S., Simon H.U. (2019). Regulation of the innate immune system by autophagy: Monocytes, macrophages, dendritic cells and antigen presentation. Cell Death Differ..

[386] Carrero J.A., McCarthy D.P., Ferris S.T., Wan X., Hu H., Zinselmeyer B.H., Vomund A.N., Unanue E.R. (2017). Resident macrophages of pancreatic islets have a seminal role in the initiation of autoimmune diabetes of NOD mice. Proc. Natl. Acad. Sci. USA.

[387] Jiang Z., Jiang J.X., Zhang G.X. (2014). Macrophages: A double-edged sword in experimental autoimmune encephalomyelitis. Immunol. Lett..

[388] Funes S.C., Rios M., Escobar-Vera J., Kalergis A.M. (2018). Implications of macrophage polarization in autoimmunity. Immunology.

[389] Weitz J.R., Jacques-Silva C., Qadir M.M.F., Umland O., Pereira E., Qureshi F., Tamayo A., Dominguez-Bendala J., Rodriguez-Diaz R., Almaça J. (2020). Secretory Functions of Macrophages in the Human Pancreatic Islet Are Regulated by Endogenous Purinergic Signaling. Diabetes.

[390] Wong P.F., Wei W., Gupta S., Smithy J.W., Zelterman D., Kluger H.M., Rimm D.L. (2019). Multiplex quantitative analysis of cancer-associated fibroblasts and immunotherapy outcome in metastatic melanoma. J. Immunother. Cancer.

[391] Chu F., Shi M., Zheng C., Shen D., Zhu J., Zheng X., Cui L. (2018). The roles of macrophages and microglia in multiple sclerosis and experimental autoimmune encephalomyelitis. J. Neuroimmunol..

[392] Li F., Yang Y., Zhu X., Huang L., Xu J. (2015). Macrophage Polarization Modulates Development of Systemic Lupus Erythematosus. Cell Physiol. Biochem..

[393] Parsa R., Andresen P., Gillett A., Mia S., Zhang X.M., Mayans S., Holmberg D., Harris R.A. (2012). Adoptive transfer of immunomodulatory M2 macrophages prevents type 1 diabetes in NOD mice. Diabetes.

[394] Zhong Z., Umemura A., Sanchez-Lopez E., Liang S., Shalapour S., Wong J., He F., Boassa D., Perkins G., Ali S.R. (2016). NF-κB Restricts Inflammasome Activation via Elimination of Damaged Mitochondria. Cell.

[395] Kanayama M., Inoue M., Danzaki K., Hammer G., He Y.W., Shinohara M.L. (2015). Autophagy enhances NFκB activity in specific tissue macrophages by sequestering A20 to boost antifungal immunity. Nat. Commun..

[396] Gupta N., Jadhav K., Shah V. (2017). Emperipolesis, entosis and cell cannibalism: Demystifying the cloud. J. Oral Maxillofac. Pathol..

[397] Borensztejn K., Tyrna P., Gaweł A.M., Dziuba I., Wojcik C., Bialy L.P., Mlynarczuk-Bialy I. (2021). Classification of Cell-in-Cell Structures: Different Phenomena with Similar Appearance. Cells.

[398] Fais S., Overholtzer M. (2018). Cell-in-cell phenomena, cannibalism, and autophagy: Is there a relationship?. Cell Death Dis..

[399] Miao Q., Bian Z., Tang R., Zhang H., Wang Q., Huang S., Xiao X., Shen L., Qiu D., Krawitt E.L. (2015). Emperipolesis mediated by CD8 T cells is a characteristic histopathologic feature of autoimmune hepatitis. Clin. Rev. Allergy Immunol..

[400] Chen L., Kong D., Xia S., Wang F., Li Z., Zhang F., Zheng S. (2022). Crosstalk Between Autophagy and Innate Immunity: A Pivotal Role in Hepatic Fibrosis. Front. Pharmacol..

[401] Shi J., Zhao J., Zhang X., Cheng Y., Hu J., Li Y., Zhao X., Shang Q., Sun Y., Tu B. (2017). Activated hepatic stellate cells impair NK cell anti-fibrosis capacity through a TGF-β-dependent emperipolesis in HBV cirrhotic patients. Sci. Rep..

[402] O’Sullivan T.E., Johnson L.R., Kang H.H., Sun J.C. (2015). BNIP3- and BNIP3L-Mediated Mitophagy Promotes the Generation of Natural Killer Cell Memory. Immunity.

[403] Huang P., Wang F., Yang Y., Lai W., Meng M., Wu S., Peng H., Wang L., Zhan R., Imani S. (2019). Hematopoietic-Specific Deletion of Foxo1 Promotes NK Cell Specification and Proliferation. Front. Immunol..

[404] Billis A., Assis-Mendonça G.R., Tavares T.F., Parreira K., Costa L.B.E., Barreto I.S., Freitas L.L.L. (2022). Fumarate hydratase-deficient renal cell carcinoma: A tumor with diverse morphology including cannibalism, lymphocytic emperipolesis, and defective autophagy. Ann. Diagn. Pathol..

[405] Florey O., Kim S.E., Sandoval C.P., Haynes C.M., Overholtzer M. (2011). Autophagy machinery mediates macroendocytic processing and entotic cell death by targeting single membranes. Nat. Cell Biol..

[406] Martins I., Raza S.Q., Voisin L., Dakhli H., Law F., De Jong D., Allouch A., Thoreau M., Brenner C., Deutsch E. (2017). Entosis: The emerging face of non-cell-autonomous type IV programmed death. Biomed. J..

[407] Davies S.P., Reynolds G.M., Wilkinson A.L., Li X., Rose R., Leekha M., Liu Y.S., Gandhi R., Buckroyd E., Grove J. (2019). Hepatocytes Delete Regulatory T Cells by Enclysis, a CD4+ T Cell Engulfment Process. Cell Rep..

[408] Mlynarczuk-Bialy I., Dziuba I., Sarnecka A., Platos E., Kowalczyk M., Pels K.K., Wilczynski G.M., Wojcik C., Bialy L.P. (2020). Entosis: From Cell Biology to Clinical Cancer Pathology. Cancers.

[409] Durgan J., Tseng Y.Y., Hamann J.C., Domart M.C., Collinson L., Hall A., Overholtzer M., Florey O. (2017). Mitosis can drive cell cannibalism through entosis. Elife.

[410] Kianfar M., Balcerak A., Chmielarczyk M., Tarnowski L., Grzybowska E.A. (2022). Cell Death by Entosis: Triggers, Molecular Mechanisms and Clinical Significance. Int. J. Mol. Sci..

[411] Pezzano M., Samms M., Martinez M., Guyden J. (2001). Questionable thymic nurse Cell. Microbiol. Mol. Biol. Rev..

[412] He M.F., Wang S., Wang Y., Wang X.N. (2013). Modeling cell-in-cell structure into its biological significance. Cell Death Dis..

[413] Hendrix T.M., Chilukuri R.V., Martinez M., Olushoga Z., Blake A., Brohi M., Walker C., Samms M., Guyden J.C. (2010). Thymic nurse cells exhibit epithelial progenitor phenotype and create unique extra-cytoplasmic membrane space for thymocyte selection. Cell Immunol..

[414] Palm W., Park Y., Wright K., Pavlova N.N., Tuveson D.A., Thompson C.B. (2015). The Utilization of Extracellular Proteins as Nutrients Is Suppressed by mTORC1. Cell.

[415] Krajcovic M., Krishna S., Akkari L., Joyce J.A., Overholtzer M. (2013). mTOR regulates phagosome and entotic vacuole fission. Mol. Biol. Cell..

[416] Sun L., Meng Z., Zhu Y., Lu J., Li Z., Zhao Q., Huang Y., Jiang L., Yao X. (2018). TM9SF4 is a novel factor promoting autophagic flux under amino acid starvation. Cell Death Differ..

[417] Han R., Gao J., Zhai H., Xiao J., Ding Y., Hao J. (2016). RAD001 (everolimus) attenuates experimental autoimmune neuritis by inhibiting the mTOR pathway, elevating Akt activity and polarizing M2 macrophages. Exp. Neurol..

[418] Esposito M., Ruffini F., Bellone M., Gagliani N., Battaglia M., Martino G., Furlan R. (2010). Rapamycin inhibits relapsing experimental autoimmune encephalomyelitis by both effector and regulatory T cells modulation. J. Neuroimmunol..

[419] Prevel N., Allenbach Y., Klatzmann D., Salomon B., Benveniste O. (2013). Beneficial role of rapamycin in experimental autoimmune myositis. PLoS ONE.

[420] Maeda K., Shioi T., Kosugi R., Yoshida Y., Takahashi K., Machida Y., Izumi T. (2005). Rapamycin ameliorates experimental autoimmune myocarditis. Int. Heart J..

[421] Yoshida Y., Shioi T., Izumi T. (2007). Resveratrol ameliorates experimental autoimmune myocarditis. Circ. J..

[422] Sato F., Martinez N.E., Shahid M., Rose J.W., Carlson N.G., Tsunoda I. (2013). Resveratrol exacerbates both autoimmune and viral models of multiple sclerosis. Am. J. Pathol..

[423] Kinoshita K., Yoo B.S., Nozaki Y., Sugiyama M., Ikoma S., Ohno M., Funauchi M., Kanamaru A. (2003). Retinoic acid reduces autoimmune renal injury and increases survival in NZB/W F1 mice. J. Immunol..

[424] Hennig M., Bauer D., Wasmuth S., Busch M., Walscheid K., Thanos S., Heiligenhaus A. (2012). Everolimus improves experimental autoimmune uveoretinitis. Exp. Eye Res..

[425] Guo X., Harada C., Namekata K., Kimura A., Mitamura Y., Yoshida H., Matsumoto Y., Harada T. (2011). Spermidine alleviates severity of murine experimental autoimmune encephalomyelitis. Invest. Ophthalmol. Vis. Sci..

[426] Chiuso-Minicucci F., Ishikawa L.L., Mimura L.A., Fraga-Silva T.F., França T.G., Zorzella-Pezavento S.F., Marques C., Ikoma M.R., Sartori A. (2015). Treatment with Vitamin D/MOG Association Suppresses Experimental Autoimmune Encephalomyelitis. PLoS ONE.

[427] Thomé R., Moraes A.S., Bombeiro A.L., Farias Ados S., Francelin C., da Costa T.A., Di Gangi R., dos Santos L.M., de Oliveira A.L., Verinaud L. (2013). Chloroquine treatment enhances regulatory T cells and reduces the severity of experimental autoimmune encephalomyelitis. PLoS ONE.

[428] Ma X., Dai Y., Witzke O., Xu S., Lindemann M., Kribben A., Dolff S., Wilde B. (2022). Chloroquine Suppresses Effector B-Cell Functions and Has Differential Impact on Regulatory B-Cell Subsets. Front. Immunol..

[429] Grandér D., Kharaziha P., Laane E., Pokrovskaja K., Panaretakis T. (2009). Autophagy as the main means of cytotoxicity by glucocorticoids in hematological malignancies. Autophagy.

[430] Wang Y., Liu F., Fang C., Xu L., Chen L., Xu Z., Chen J., Peng W., Fu B., Li Y. (2021). Combination of rapamycin and SAHA enhanced radiosensitization by inducing autophagy and acetylation in NSCLC. Aging.

[431] Sang J., Gan L., Zou M.F., Lin Z.J., Fan R.Z., Huang J.L., Li W., Tang G.H., Yin S. (2022). Jolkinolide B sensitizes bladder cancer to mTOR inhibitors via dual inhibition of Akt signaling and autophagy. Cancer Lett..

[432] Fatehi R., Rashedinia M., Akbarizadeh A.R., Zamani M., Firouzabadi N. (2023). Metformin enhances anti-cancer properties of resveratrol in MCF-7 breast cancer cells via induction of apoptosis, autophagy and alteration in cell cycle distribution. Biochem. Biophys. Res. Commun..

[433] Fukuda M., Ogasawara Y., Hayashi H., Inoue K., Sakashita H. (2022). Resveratrol Inhibits Proliferation and Induces Autophagy by Blocking SREBP1 Expression in Oral Cancer Cells. Molecules.

[434] Moosavi M.A., Djavaheri-Mergny M. (2019). Autophagy: New Insights into Mechanisms of Action and Resistance of Treatment in Acute Promyelocytic leukemia. Int. J. Mol. Sci..

[435] Bhutia S.K. (2022). Vitamin D in autophagy signaling for health and diseases: Insights on potential mechanisms and future perspectives. J. Nutr. Biochem..

[436] Rahman M.A., Saikat A.S.M., Rahman M.S., Islam M., Parvez M.A.K., Kim B. (2023). Recent Update and Drug Target in Molecular and Pharmacological Insights into Autophagy Modulation in Cancer Treatment and Future Progress. Cells.

[437] Jiang T., Chen X., Ren X., Yang J.M., Cheng Y. (2021). Emerging role of autophagy in anti-tumor immunity: Implications for the modulation of immunotherapy resistance. Drug Resist. Updat..

[438] Wahba J., Natoli M., Whilding L.M., Parente-Pereira A.C., Jung Y., Zona S., Lam E.W., Smith J.R., Maher J., Ghaem-Maghami S. (2018). Chemotherapy-induced apoptosis, autophagy and cell cycle arrest are key drivers of synergy in chemo-immunotherapy of epithelial ovarian cancer. Cancer Immunol. Immunother..

[439] Zhang Q., Lu W., Liang C.L., Chen Y., Liu H., Qiu F., Dai Z. (2018). Chimeric Antigen Receptor (CAR) Treg: A Promising Approach to Inducing Immunological Tolerance. Front. Immunol..

[440] Gliwiński M., Iwaszkiewicz-Grześ D., Wołoszyn-Durkiewicz A., Tarnowska M., Żalińska M., Hennig M., Zielińska H., Dukat-Mazurek A., Zielkowska-Dębska J., Zieliński M. (2020). Proinsulin-specific T regulatory cells may control immune responses in type 1 diabetes: Implications for adoptive therapy. BMJ Open Diabetes Res. Care.

[441] Zhang C., Sun Y., Li S., Shen L., Teng X., Xiao Y., Wu N., Lu Z. (2022). Autophagic flux restoration enhances the antitumor efficacy of tumor infiltrating lymphocytes. J. Immunother. Cancer.

[442] Lu Y., Gao J., Zhang S., Gu J., Lu H., Xia Y., Zhu Q., Qian X., Zhang F., Zhang C. (2018). miR-142–3p regulates autophagy by targeting ATG16L1 in thymic-derived regulatory T cell (tTreg). Cell Death Dis..

[443] Jang A., Sharp R., Wang J.M., Feng Y., Wang J., Chen M. (2021). Dependence on Autophagy for Autoreactive Memory B Cells in the Development of Pristane-Induced Lupus. Front. Immunol..

[444] Keller C.W., Sina C., Kotur M.B., Ramelli G., Mundt S., Quast I., Ligeon L.-A., Weber P., Becher B., Münz C. (2017). ATG-dependent phagocytosis in dendritic cells drives myelin-specific CD4+ T cell pathogenicity during CNS inflammation. Proc. Natl. Acad. Sci. USA.

[445] Johnson D.B., Beckermann K.E., Wang D.Y. (2018). Immune Checkpoint Inhibitor Therapy in Patients With Autoimmune Disease. Oncology.

